# ADAMTS5 Is a Critical Regulator of Virus-Specific T Cell Immunity

**DOI:** 10.1371/journal.pbio.1002580

**Published:** 2016-11-17

**Authors:** Meagan McMahon, Siying Ye, Leonard Izzard, Daniel Dlugolenski, Ralph A. Tripp, Andrew G. D. Bean, Daniel R. McCulloch, John Stambas

**Affiliations:** 1 School of Medicine, Deakin University, Waurn Ponds, Victoria, Australia; 2 College of Veterinary Medicine, University of Georgia, Athens, Georgia, United States of America; 3 Australian Animal Health Laboratory, CSIRO, East Geelong, Victoria, Australia; National Jewish Medical and Research Center/Howard Hughes Medical Institute, UNITED STATES

## Abstract

The extracellular matrix (ECM) provides physical scaffolding for cellular constituents and initiates biochemical and biomechanical cues that are required for physiological activity of living tissues. The ECM enzyme ADAMTS5, a member of the ADAMTS (A Disintegrin-like and Metalloproteinase with Thrombospondin-1 motifs) protein family, cleaves large proteoglycans such as aggrecan, leading to the destruction of cartilage and osteoarthritis. However, its contribution to viral pathogenesis and immunity is currently undefined. Here, we use a combination of in vitro and in vivo models to show that ADAMTS5 enzymatic activity plays a key role in the development of influenza-specific immunity. Influenza virus infection of *Adamts5*^*-/-*^ mice resulted in delayed virus clearance, compromised T cell migration and immunity and accumulation of versican, an ADAMTS5 proteoglycan substrate. Our research emphasises the importance of ADAMTS5 expression in the control of influenza virus infection and highlights the potential for development of ADAMTS5-based therapeutic strategies to reduce morbidity and mortality.

## Abstract 2

The extracellular matrix enzyme ADAMTS5 enhances the clearance of viruses by facilitating migration of T lymphocytes to the periphery following influenza virus infection.

## Author Summary

Movement of immune cells is critical for effective clearance of pathogens. The response to influenza virus infection requires immune cell trafficking between the lung, mediastinal lymph node and other peripheral lymphoid organs such as the spleen. We set out to assess the contribution of a specific extracellular matrix enzyme, ADAMTS5, to migration of lymphocytes and overall pathogenesis following infection. In our studies, we demonstrate that mice lacking *Adamts5* have fewer influenza-specific lymphocytes in the lung and spleen following infection. These observations correlated with an accumulation of influenza-specific lymphocytes in the mediastinal lymph node and increased virus titres. This work suggests that ADAMTS5 is necessary for immune cell migration to the periphery, where lymphocyte function is required to fight infection.

## Introduction

Influenza A virus infection is responsible for substantial global morbidity and mortality (>500,000 deaths each year [1]) and largely afflicts high-risk groups, including the very young and elderly. There are currently two countermeasures employed to control influenza virus infection: vaccines and antivirals. Although generally effective, the imperfect proofreading capacity of the RNA-dependent RNA polymerase drives constant genetic drift. Moreover, a segmented genome facilitates rapid genetic shift, resulting in the need for reformulation of seasonal vaccines or the emergence of resistance following administration of antivirals, leading to suboptimal prophylactic or therapeutic intervention [2].

T cells are a vital component of the adaptive immune response following influenza virus infection. Critically, trafficking of activated influenza-specific T cells from draining lymph nodes (including the mediastinal lymph node [MLN]) to the site of primary infection in the lung requires direct contact and interaction with the extracellular matrix (ECM) [3]. The ECM provides adhesive substrates, such as proteoglycans and collagen, to encourage and facilitate lymphocyte trafficking [4]. Expression and remodelling of ECM components is strictly regulated to control movement of immune cells. Therefore, it is not surprising that perturbations in substrate availability and ECM remodelling significantly impact granulocyte and lymphocyte migration in a number of model systems [5–7].

The A Disintegrin-like and Metalloproteinase with Thrombospondin-1 motifs (ADAMTS) family are a group of secreted metalloproteinases found within the zinc-dependent metzincin super-family that also consists of matrix metalloproteinases (MMPs) and ADAMs [8]. The ADAMTS family comprises 19 mammalian ADAMTs enzymes [9]. ADAMTS5 is one of the most highly characterised and well-known proteinases in this family and has been shown to cleave the hyalectan class of chondroitin sulphate proteoglycans (CSPGs), including aggrecan, brevican, neurocan, and versican [10–13]. Hyalectans/CSPGs are large aggregating macromolecules that hydrate tissue and confer rigidity to the extracellular space. ADAMTS5 has become a major drug target for arthritis therapy as ADAMTS5 knockout mice (*Adamts5*^*-/-*^ mice) are resistant to aggrecan cleavage in articular cartilage and are thus protected from experimentally induced arthritis [14,15]. Aside from the documented role in arthritis, ADAMTS5 has been shown to play a role in embryonic development, including limb and cardiac morphogenesis, and skeletal muscle development through its versican remodelling properties [11,16,17]. Importantly, its role in viral immunity is currently undefined.

Versican, a substrate of ADAMTS5, is a widely expressed tissue proteoglycan involved in cell adhesion, proliferation, and migration [4]. The two predominant splice-variants of versican that harbour ADAMTS cleavage sites in their shared glycosaminoglycan (GAG)-β domain are V0 and V1 [18]. GAG chains provide interactive points for antigen recognition receptors (Toll-like receptor 2 and 4), chemokines (MCP-1, MCP-2, CCL5), and cell surface markers (CD62L, CD44), some of which are directly linked to immune cell migration [19–21]. Furthermore, in vitro studies have shown that Poly I:C induced versican expression can restrict CD4^+^ T cell migration by preventing ECM adhesion [22]. Given the fact that ADAMTS5 is a versicanase [11], we hypothesised that it would play a key role in viral immunity. Our data demonstrates that host expression of ADAMTS5 is required to help ameliorate disease following influenza virus infection. *Adamts5*^*-/-*^ mice clearly show increased weight loss and higher viral titres throughout the course of influenza virus infection along with impaired CD8^+^ T cell migration and immunity.

## Results

### Phenotyping of *Adamts5*^*-/-*^ Immune Cells

**Fig 1:**
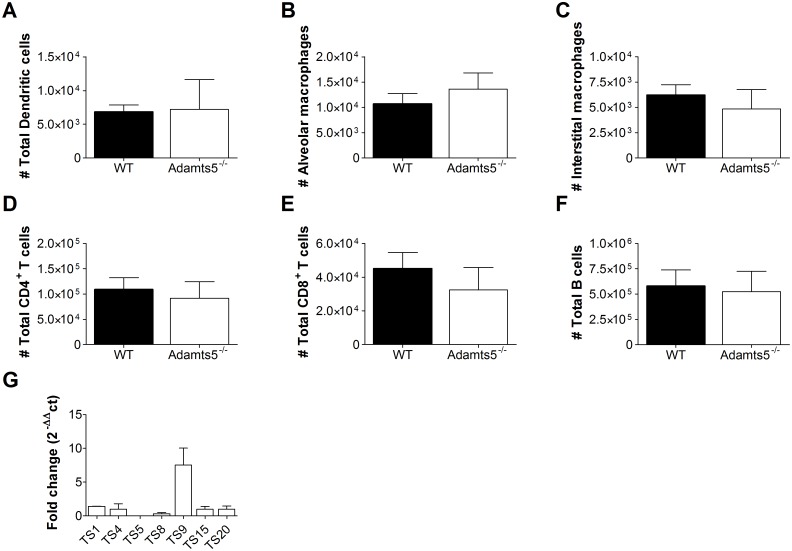
Immune cell subsets in naïve *Adamts5*^*-/-*^ mice. Spleens and lungs from naïve C57.BL/6 and *Adamts5*^*-/-*^ mice were removed and immune cell subsets and ADAMTS expression levels characterised. **(A)** Dendritic cells (CD11c^+^MHCII^+^), **(B)** alveolar macrophages (CD11c^+^F4/80^+^), and **(C)** interstitial macrophages (CD11b^+^F4/80^+^) in the lung, and **(D)** CD4^+^ and **(E)** CD8^+^ T cells and **(F)** B cells (B220^+^) in the spleen are shown. **(G)** Basal expression levels of ADAMTS enzymes in lung of naïve C57.BL/6 and *Adamts5*^*-/-*^ mice, with the data normalised to naïve C57.Bl/6 controls. WT denotes C57.BL/6 mice. Results are expressed as means ± SD, and statistical significance (*p* < 0.05 relative to C57.BL/6) determined by Student’s *t* test (*n* = 5 representing three experiments). Underlying data are provided in S1 Data.

ADAMTS enzymes are widely distributed in human adult tissues and play a key role in normal cellular function. Although *Adamts5*^*-/-*^ mice are viable and phenotypically normal, based on gross analysis of histological samples [14,15], detailed characterisation has revealed decreased interdigital web regression leading to fused digits [11], cardiac valve maturation [17], and abnormal formation of multinucleated myotubes required for skeletal muscle development [16]. In contrast, the homeostatic immune cell composition of naïve *Adamts5*^*-/-*^ mice has yet to be determined. Antibody staining and flow cytometric analysis of immune cell populations performed prior to infection suggested that the proportion of dendritic cells (CD11c^+^MHCII^+^), alveolar macrophages (CD11c^+^F480^+^), and interstitial macrophages (CD11b^+^F480^+^) were comparable in the lungs of C57.BL/6 (wild-type [WT] control mice) and *Adamts5*^-/-^ mice (Fig 1A–1C), as were the number of CD4^+^ and CD8^+^ T lymphocytes and B cells in the spleen (Fig 1D–1F). Furthermore, we analysed immune cell populations (dendritic cells, macrophages, NK, T and B cells) in the lung (S1 Fig), spleen (S2 Fig), and thymus (S3 Fig) and found no observable differences between *Adamts5*^*-/-*^ mice and C57.BL/6 controls. As such, we concluded that *Adamts5*^-/-^ mice were immunologically “normal” prior to infection.

We also carefully analysed the gene expression level of related ADAMTS family members that have “versicanase” activity to determine if compensation of enzymatic activity was evident. Quantitative reverse-transcriptase polymerase chain reaction (QRT-PCR) data demonstrated similar gene expression levels of *Adamts1*, *4*, *8*, *15*, and *20* in the lungs of WT C57.BL/6 and *Adamts5*^*-/-*^ mice (Fig 1G). However, increased expression of the *Adamts9* versicanase was observed in *Adamts5*^*-/-*^ mice when compared to WT controls, although this was not statistically significant (*p* = 0.067).

### *Adamts5*^*-/-*^ Mice Exhibit Increased Weight Loss and Delayed Influenza Virus Clearance following Infection

**Fig 2:**
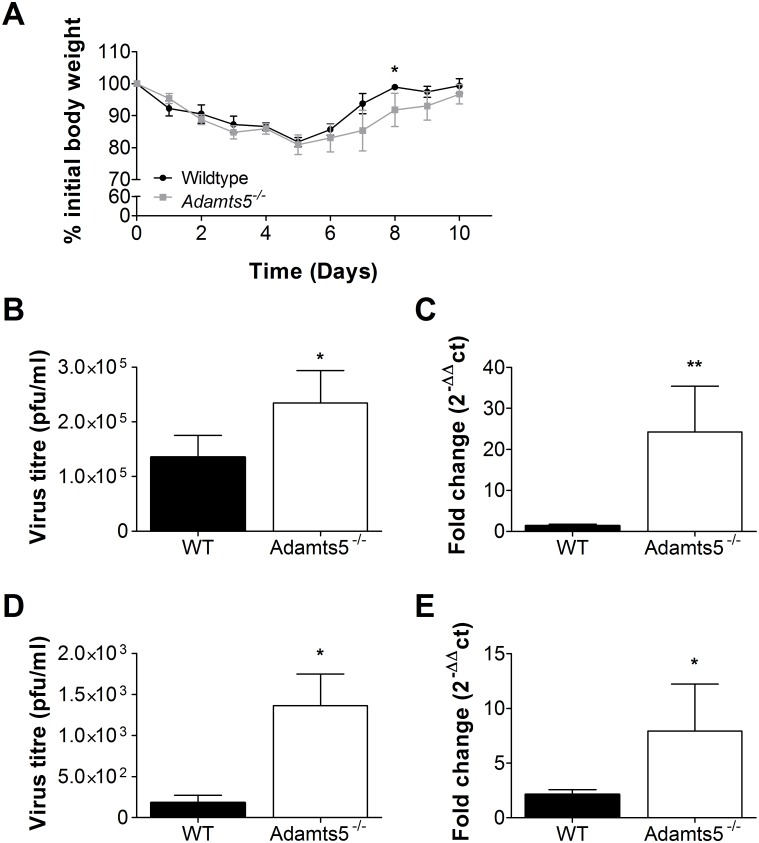
Lack of ADAMTS5 expression in *Adamts5*^*-/-*^ mice results in increased weight loss and delayed virus clearance. C57.BL/6 and *Adamts5*^*-/-*^ mice were intranasally infected with 10^4^ pfu X31 (H3N2) influenza virus. **(A)** Weight loss over a 10-d period of infection was observed. Virus titres were determined from day 3 and day 7 lung homogenates using a **(B** and **D)** plaque assay and **(C** and **E)** qRT-PCR of Matrix-1 gene amplification. WT denotes C57.BL/6 mice. The results are expressed as means ± SD, and statistical significance (relative to C57.BL/6 controls) was determined by Student’s *t* test (* *p* ≤ 0.05, **p ≤ 0.01, *n* = 5 representing three experiments). Underlying data are provided in S1 Data.

To investigate the role of ADAMTS5 in influenza virus infection, we initially examined in vivo weight loss and lung virus replication kinetics following infection. Influenza virus titres normally peak 3 d post infection (p.i.), and virus is cleared by day 7–10 p.i. [23]. We intranasally infected *Adamts5*^*-/-*^ mice and C57.BL/6 controls with 10^4^ pfu/mouse-adapted X31 (H3N2) influenza virus, and observed enhanced weight loss (*p* < 0.05 on day 8 p.i.) in *Adamts5*^*-/-*^ mice across the experimental infection period when compared to C57.BL/6 controls (Fig 2A). At the peak of viremia (day 3 p.i.), *Adamts5*^*-/-*^ mice showed higher virus titres in the lung when compared to C57.BL/6 controls by both plaque assay (Fig 2B) and QRT-PCR analysis of influenza virus Matrix-1 gene expression (Fig 2C). Similar observations were recorded at 7 d p.i. (Fig 2D and 2E), suggesting *Adamts5*^*-/-*^ mice do not clear influenza virus as effectively as C57.BL/6 controls. Additionally, a qPCR time-course analysis of ADAMTS enzyme expression in the lungs of influenza-infected WT and *Adamts5*^*-/-*^ mice was also performed and shown in S4 Fig for general reference.

### Influenza-Specific Cellular Immunity in *Adamts5*^*-/-*^ Knockout Mice

**Fig 3:**
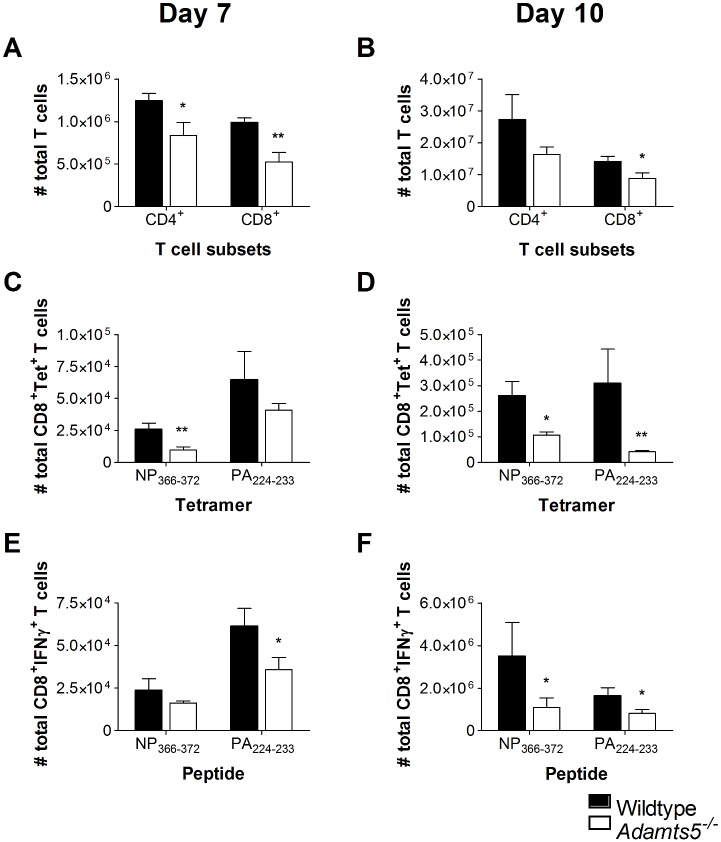
Reduced CD4^+^ and CD8^+^ T cell numbers in the spleens of influenza-infected *Adamts5*^*-/-*^ mice. C57.BL/6 and *Adamts5*^*-/-*^ mice were intranasally (i.n.) infected (10^4^ pfu/mouse) with X31 (H3N2) influenza virus. Spleens were removed at day 7 and day 10 p.i., and CD8^+^ T cell responses determined. Total CD4^+^ and CD8^+^ T cells numbers were calculated at days **(A)** 7 and **(B)** 10 p.i. Influenza-specific D^b^NP_366-372_ CD8^+^ and D^b^PA_224-233_ CD8^+^ tetramer-positive T cell numbers were measured at days **(C)** 7 and **(D)** 10 p.i. Functional influenza-specific D^b^NP_366-372_^+^IFNγ^+^CD8^+^ and D^b^PA_224-233_^+^IFNγ^+^CD8^+^ T cell activity was determined by ICS, and total IFNγ^+^ T cells enumerated at days **(E)** 7 and **(F)** 10 after infection. WT denotes C57.BL/6. The results are expressed as means ± SD, and statistical significance (relative to C57.BL/6) was determined by Student’s *t* test (*p ≤ 0.05, **p ≤ 0.01, *n* = 5 representing three experiments). Underlying data are provided in S1 Data.

**Fig 4:**
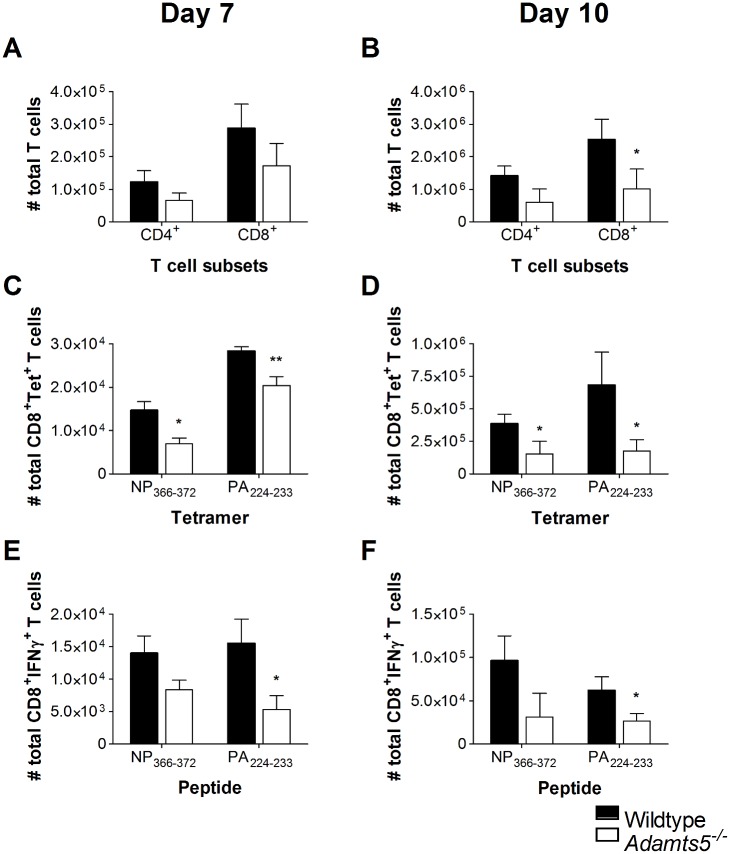
CD4^+^ and CD8^+^ T cell responses in the lungs of influenza-infected *Adamts5*^*-/-*^ mice. C57.BL/6 and *Adamts5*^*-/-*^ mice were infected i.n. with 10^4^pfu X31 (H3N2) influenza virus. Lungs were removed at days 7 and 10 p.i., and CD8^+^ T cell immunity characterised. Total CD4^+^ and CD8^+^ T cell numbers are shown at days **(A)** 7 and **(B)** 10 p.i. Influenza-specific tetramer^+^ D^b^NP_366-372_^+^ CD8^+^ and D^b^PA_224-233_^+^ CD8^+^ T cell responses were enumerated at days **(C)** 7 and **(D)** 10 p.i. CD8^+^ T cell functionality was assessed by ICS and D^b^NP_366-372_^+^IFNγ^+^CD8^+^ and D^b^PA_224-233_^+^IFNγ^+^CD8^+^ T cell responses enumerated at days **(E)** 7 and **(F)** 10 p.i. Wildtype denotes C57.BL/6 mice. The results are expressed as means ± SD, and statistical significance (relative to C57.BL/6) was determined by Student’s *t* test (* = *p* ≤ 0.05, ** = *p ≤* 0.01, *n* = 5 representing three experiments). Underlying data are provided in S1 Data.

**Fig 5:**
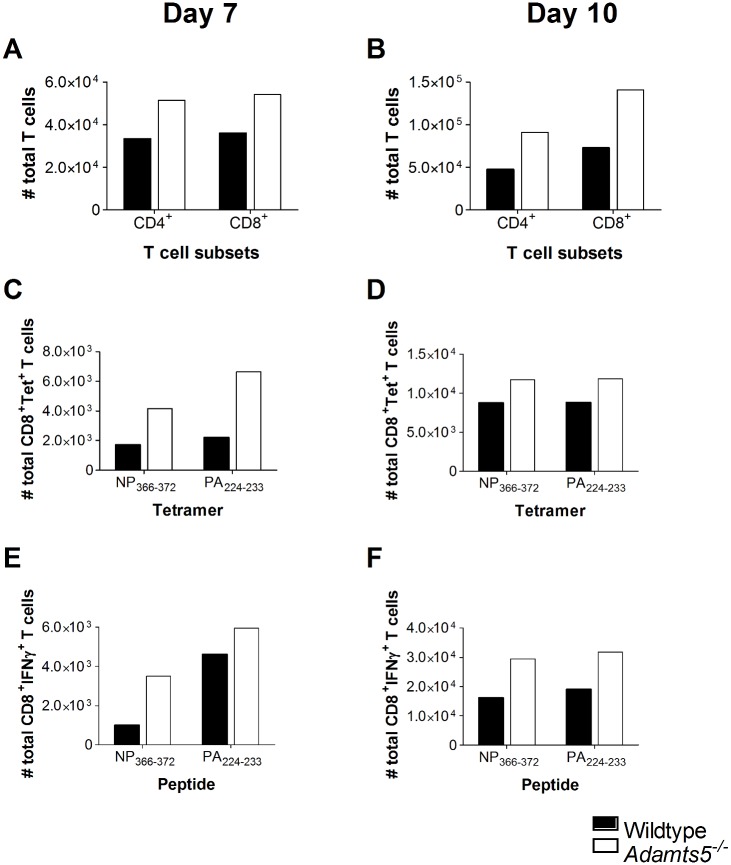
T cell immunity in the pooled MLN. C57.BL/6 and *Adamts5*^*-/-*^ mice were infected i.n. with 10^4^ pfu/mouse X31 (H3N2) influenza virus. MLNs were removed, pooled, and processed at days 7 and 10 p.i., and single-cell suspensions analysed for influenza-specific immunity. Total CD4^+^ and CD8^+^ T cell numbers were determined at days **(A)** 7 and **(B)** 10 p.i. Influenza-specific D^b^NP_366-372_^+^ CD8^+^ and D^b^PA_224-233_^+^ CD8^+^ tetramer positive T cells were enumerated at days **(C)** 7 and **(D)** 10 p.i. CD8^+^ T cell functionality was measured using ICS. Influenza-specific D^b^NP_366-372_^+^IFNγ^+^CD8^+^ and D^b^PA_224-233_^+^IFNγ^+^CD8^+^ T cell responses were characterised at days **(E)** 7 and **(F)** 10 p.i. Results are expressed as total pooled means from five mice repeated three times. Wildtype denotes C57.BL/6 mice. Underlying data are provided in S1 Data.

As there was evidence of delayed viral clearance in *Adamts5*^*-/-*^ knockout mice, we set out to determine if this observation was associated with perturbations in cellular immunity. We initially enumerated total CD4^+^ and CD8^+^ T cell numbers in the lungs and spleens of *Adamts5*^*-/-*^ and C57.BL/6 control mice to determine if the delay in viral clearance observed in Fig 2 correlated with functional differences in T cell populations. We observed decreased numbers of total CD4^+^ and CD8^+^ T cells in the spleen and lung of *Adamts5*^*-/-*^ mice at days 7 and 10 following infection (Figs 3A, 3B, 4A and 4B). In the C57.BL/6 mouse model of influenza virus infection, influenza-specific CD8^+^ T cell immunity is first detected 4–5 d p.i. and peaks at day 10 p.i. [23,24]. In our study, influenza-specific CD8^+^ T cells were enumerated using tetrameric complexes that recognised the immunodominant D^b^NP_366-372_ (ASNENMETM) or D^b^PA_224-233_ (SSLENFRAYV) CD8^+^ T cell epitopes [25]. Fewer D^b^NP_366-372_ and D^b^PA_224-233_ CD8^+^ T cells were detected in the spleen and lung of *Adamts5*^*-/-*^ mice at both day 7 and 10 p.i. when compared to C57.BL/6 controls (Figs 3C, 3D, 4C and 4D). The intracellular cytokine staining (ICS) assay was then used to assess the functionality of the CD8^+^ T cell response in the spleen and lung at multiple time points following infection. *Adamts5*^*-/-*^ mice had fewer D^b^NP_366-372_^+^IFNγ^+^CD8^+^ and D^b^PA_224-233_^+^IFNγ^+^CD8^+^ T cells in the spleen and lung at days 7 and 10 p.i. (Figs 3E, 3F, 4E and 4F). The lack of influenza-specific CD8^+^ T cells in the periphery of *Adamts5*^*-/-*^ mice suggested possible accumulation of cells in the draining lymph nodes of the lung, such as the MLN. Careful analysis revealed increased numbers of total CD4^+^ and CD8^+^ T cells and D^b^NP_366-372_^+^ and D^b^PA_224-233_^+^ tetramer^+^ CD8^+^ T cells in the pooled MLN of *Adamts5* ^*-/-*^ mice when compared to controls 7 and 10 d p.i. (Fig 5A–5D). These observations were further validated using ICS of pooled MLN (Fig 5E and 5F) and suggested that the ECM remodelling by ADAMTS5 contributes to migration of effector T cells from the MLN to peripheral tissue.

### Accumulation of Versican in the MLN of *Adamts5*^*-/-*^ Mice

**Fig 6:**
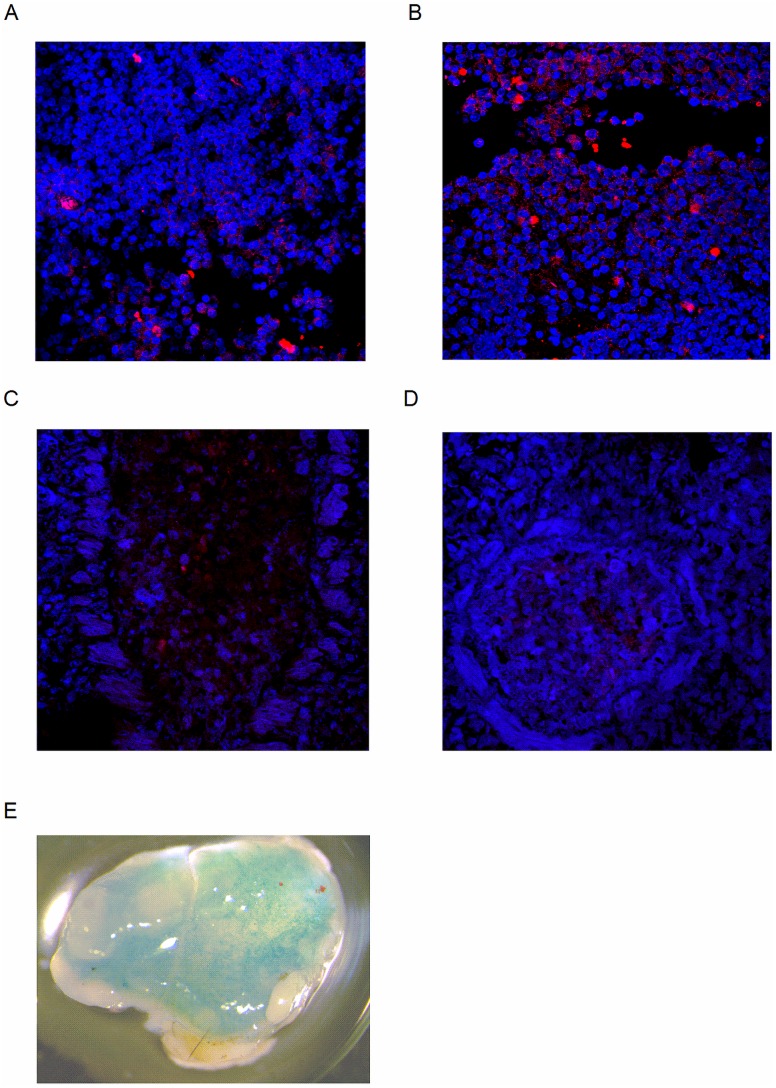
Versican accumulation in MLN prevents T cell egress. C57.BL/6 and *Adamts5*^*-/-*^ mice were i.n. infected (10^4^ pfu/mouse) with X31 (H3N2) influenza virus. MLNs were removed, sectioned, and stained for expression of versican (GAGβ) and versikine (DPEAAE). Versican staining in the MLN of **(A)** C57.BL/6 and **(B)** *Adamts5*^*-/-*^ mice was assessed 7 d p.i. Blue = DAPI, Red = versican (GAGβ) (*n* = 5). Versikine (DPEAAE fragment) in the MLN of **(C)** C57.BL/6 and **(D)** *Adamts5*^*-/-*^ mice was also assessed at 7 days p.i. Scale Bar = 10 μm. Blue = DAPI, Red = versikine (DPEAAE) (*n* = 5). **(E)** ADAMTS5 expression in the MLN was determined using X-gal staining for the Lac-Z reporter gene present in *Adamts5*^*-/-*^ mice. (*n* = 3 representing three separate experiments).

ADAMTS5 is an important enzyme involved in the remodelling of the ECM, and its actions are thought to contribute to the trafficking of key immune cell populations, such as macrophages [26]. While the hyalectans (aggrecan, brevican, and neurocan) are tissue specific, V0/V1 versican isoforms are widely expressed throughout the body [4,27]. We therefore reasoned that the lack of ADAMTS5 enzymatic activity in the MLN of *Adamts5*^*-/-*^ mice would result in an accumulation of the versican substrate. It is also important to note here that the presence of versican has previously been associated with inhibition of lymphocyte migration [22,28] and may result in T cell accumulation in the MLN (Fig 5A). Moreover, versican upregulation has also been associated with inflammatory stimuli [4,29]. As such, versican and versican cleavage fragment (versikine) expression in the MLN of influenza virus infected *Adamts5*^*-/-*^ mice was assessed by immunohistochemistry. MLN tissue from infected animals was paraffin embedded, sectioned, and stained with anti-GAGβ (V0/V1 versican side chains) and anti-DPEAAE (versikine) antibodies to define expression. Confocal microscopy of sections revealed increased levels of versican in the MLN of *Adamts5*^*-/-*^ mice when compared to C57.BL/6 controls (Fig 6A and 6B). Our data also suggested that versican was expressed within the T cell areas of the lymph node following immunohistochemical staining as T cells and versican co-localised in the MLN (S5 Fig). In support of this data, decreased versikine was observed in the MLN of *Adamts5*^*-/-*^ when compared to C57.BL/6 controls (Fig 6C and 6D). *Adamts5*^*-/-*^ mice used in these studies were generated via the insertion of a Lac-Z allele into the catalytic site of the ADAMTS5 gene, and so Lac-Z expression can be used as a surrogate reporter for ADAMTS5 expression. X-gal staining of the MLN of *Adamts5*^*-/-*^ mice showed expression throughout the organ (Fig 6E). Additionally, we stained influenza-infected lung with antibodies specific for versican and versikine. Bronchioles (S6A Fig) and arteries (S6B Fig) in the lung were stained with DAPI and anti-Gagβ (versican) to compare versican expression and cleavage to that found in the MLN. No differences were observed in staining between C57.BL/6 and *Adamts5*^*-/-*^ mice (S6 Fig). This suggests that the absence of ADAMTS5 in the MLN prevents efficient cleavage of versican and results in accumulation of T cells in the draining lymph node. In contrast, the absence of ADAMTS5 in the lung does not influence versican cleavage.

### ADAMTS5-Mediated Cleavage of Versican is Necessary for T Cell Migration

**Fig 7:**
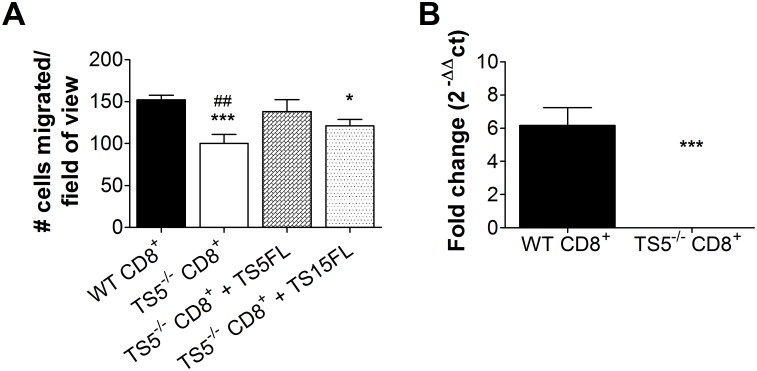
ADAMTS5 mediated cleavage of versican contributes to T cell migration. CD8^+^ T cells were magnetically enriched from the spleens of influenza virus-infected *Adamts5*^*-/-*^ and C57.BL/6 control mice, and migration of CD8^+^ T cells using a versican overlay transwell system was assessed. **(A)** *Adamts5*^*-/-*^ and C57.BL/6 CD8^+^ T cell migration through a versican overlay is shown by graphical representation. Versicanase-conditioned media was added to the upper wells of CD8^+^ T cells isolated from *Adamts5*^*-/-*^ mice. TS5FL = active ADAMTS5, TS15FL = active ADAMTS15. **(B)** ADAMTS5 expression in CD8^+^ T cells was confirmed via qRT-PCR. WT denotes C57.BL/6 mice. The results are expressed as means ± SD, and statistical significance (relative to C57.BL/6 mice) was determined by one-way ANOVA (*p ≤ 0.05, ***p ≤ 0.005 relative to CD8^+^WT, **p ≤ 0.005 relative to CD8^+^WT, ##*p ≤* 0.01 relative to CD8^+^TS5^-/-^; *n* = 3 representing three individual experiments). Underlying data are provided in S1 Data.

Given the accumulation of versican in the MLN of *Adamts5*^*-/-*^ influenza virus infected mice (Fig 6), we examined if the absence of ECM remodelling was linked to impaired CD8^+^ T cell migration. Ex vivo transwell assays were employed to assess migration of CD8^+^ T cells as previously described [30,31]. The surface of the upper transwell chamber was initially coated with versican-enriched conditioned media from transfected HEK293T cells [16,32], prior to the addition of a T cell chemoattractant (CXCL12) to the lower transwell chamber to encourage T cell migration. Enriched CD8^+^ T cells from influenza virus infected *Adamts5*^*-/-*^ or C57.BL/6 mice were then added to the upper chamber of the transwell and migration assessed. The data clearly demonstrates (*p* < 0.05) that CD8^+^ T cells isolated from *Adamts5*^*-/-*^ influenza virus infected mice show impaired migratory capacity through a versican-overlay transwell system when compared to C57.BL/6 controls expressing functional ADAMTS5 enzyme (Fig 7A). Furthermore, the introduction of exogenous versicanase (ADAMTS5/ADAMTS15 conditioned media from HEK293T cells) resulted in improved migration of CD8^+^ T cells through the versican overlay (Fig 7A). We also assessed if we could replicate these observations using human T cells. In these assays, we assessed the migration of a human immortalised CD4^+^ T cell line (JURKAT cells) following inhibition of ADAMTS5 with antibody. The data demonstrates that ADAMTS5-inhibited JURKAT cells do not migrate as efficiently as their uninhibited counterparts (S7C Fig).

We also determined that CD8^+^ T cells from C57.BL/6 mice express ADAMTS5 using qRT-PCR (Fig 7B). The expression of other versicanases (ADAMTS1, 4, 9, 15) in CD8^+^ T cells extracted from C57.BL/6 and *Adamts5*^*-/-*^ mice was also assessed and showed increased expression of ADAMTS4, 9, and 15 following influenza virus infection (S8 Fig). We also confirmed the expression of ADAMTS versicanases in JURKAT cells and peripheral blood lymphocytes (S7A and S7B Fig) and found that ADAMTS5 and 15 are highly expressed in both cell types.

Additionally, we assessed the ability of CD8^+^ T cells from C57.BL/6 and *Adamts5*^*-/-*^ mice to cleave versican. Our data indicates that versican was not cleaved as effectively by CD8^+^ T cells from *Adamts5*^*-/-*^ mice (S9 Fig). These results indicate that ADAMTS5 is indeed expressed by CD8^+^ T cells and establishes that ADAMTS5-mediated cleavage of versican is necessary for T cell migration.

### Reduced Expression of Versican in *Adamts5*^*-/-*^*Vcan* ^*+/hdf*^ Mice Restores Normal T Cell Function

**Fig 8:**
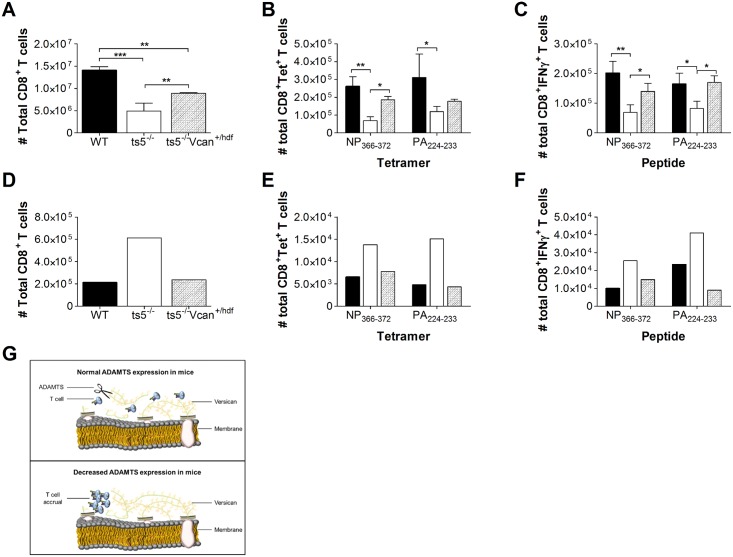
Versican reduction in *Adamts5*^*-/-*^*Vcan*^*+/hdf*^ mice restores normal T cell function. Spleen and MLNs were removed from C57.BL/6, *Adamts5*^*-/-*^*Vcan*^*+/+*^, and *Adamts5*^*-/-*^*Vcan*^*+/hdf*^ mice and processed at day 10 p.i., and single cell suspensions were analysed for influenza-specific immunity. **(A)** Total CD8^+^ T cell numbers were determined at day 10 p.i. in the spleen. **(B)** Influenza-specific D^b^NP_366-372_^+^ CD8^+^ and D^b^PA_224-233_^+^ CD8^+^ tetramer positive T cells in the spleen were enumerated at day 10 p.i. CD8^+^ T cell functionality was measured using ICS. **(C)** Influenza specific D^b^NP_366-372_^+^IFNγ^+^CD8^+^ and D^b^PA_224-233_^+^IFNγ^+^CD8^+^ T cell responses were characterised in the spleen at days 10 p.i. **(D)** Total CD8^+^ T cell numbers were assessed at day 10 p.i. in pooled MLN. **(E)** Influenza-specific D^b^NP_366-372_^+^ CD8^+^ and D^b^PA_224-233_^+^ CD8^+^ tetramer positive T cells in the pooled MLN were enumerated at day 10 p.i. CD8^+^ T cell functionality was measured using ICS. **(F)** Influenza-specific D^b^NP_366-372_^+^IFNγ^+^CD8^+^ and D^b^PA_224-233_^+^IFNγ^+^CD8^+^ T cell responses were characterised in the pooled MLN at day 10 p.i. The results are expressed as means ± SD (spleen data) or as pooled means (MLN data), and statistical significance (relative to C57.BL/6 mice) was determined by one-way ANOVA (*p ≤ 0.05, ***p ≤ 0.005 relative to C57.BL/6, *n* = 5 representing three individual experiments). WT denotes C57.BL/6 mice and *ts5*^*-/-*^ denotes *Adamts5*^*-/-*^. **(G)** Our model for ADAMTS5 enzyme activity and T cell migration proposes that versican can inhibit T cell effector function by acting as a physical block. Cleavage of versican by ADAMTS5 removes the ECM blockade, allowing migration (top panel). Moreover, versican accumulation in the absence of ADAMTS enzyme activity results in T cell clustering (bottom panel). Underlying data are provided in S1 Data.

Given the accumulation of versican observed in the MLN of *Adamts5*^*-/-*^ mice (Fig 6B) and inhibition of CD8^+^ T cell migration in transwell assays (Fig 7A), we wanted to assess if reducing versican expression would result in a rescue of T cell function. To achieve reduced versican levels, we crossed *Adamts5*^*-/-*^ mice with versican reduced mice (*Vcan*^*+/hdf*^). It should be noted that disruption of both versican alleles is embryonic lethal [33]. We infected C57.BL/6, *Adamts5*^*-/-*^*Vcan*^*+/hdf*^ (versican reduced), and *Adamts5*^*-/-*^*Vcan*^*+/+*^ mice with 10^4^ pfu X31 (H3N2) influenza virus and assessed CD8^+^ T cell immunity in the spleen and MLN at day 10 p.i. *Adamts5*^*-/-*^*Vcan*^*+/hdf*^ (versican reduced) mice showed increased numbers of total CD8^+^ T cells in the spleen at day 10 following infection when compared to the *Adamts5*^*-/-*^*Vcan*^*+/+*^ control group (Fig 8A). Increased influenza-specific D^b^NP_366-372_ and D^b^PA_224-233_ CD8^+^ T cell numbers were also detected by tetramer staining in the spleen of *Adamts5*^*-/-*^*Vcan*^*+/hdf*^ versican reduced) mice at day 10 p.i. when compared to *Adamts5*^*-/-*^*Vcan*^*+/+*^ controls (Fig 8B). This was also reflected in functional assays where *Adamts5*^*-/-*^*Vcan*^*+/hdf*^ (versican reduced) mice showed improved IFNγ production for both T cell specificities (Fig 8C). We also assessed CD8^+^ T cell numbers in MLN of influenza-infected *Adamts5*^*-/-*^*Vcan*^*+/hdf*^ (versican reduced) mice to determine resumption of egress. Careful analysis revealed comparable numbers of total CD8^+^ T cells and influenza-specific CD8^+^NP_366-372_^+^ and D^b^PA_224-233_^+^ (by tetramer and ICS) in the MLN of *Adamts5*^*-/-*^*Vcan*^*+/hdf*^ (versican reduced) and C57.BL6 control mice (Fig 8D–8F). We also assessed influenza-specific immunity in influenza virus-infected *Vcan*^*+/hdf*^ and C57.BL/6 mice and found that NP_366-372_-specific CD8^+^ T cells were increased in the lungs of *Vcan*^*+/hdf*^ mice (S10 Fig). Concurrently, NP_366-372_-specific CD8^+^ T cell numbers were decreased in the MLN of *Vcan*^*+/hdf*^ mice when compared to C57.BL/6 controls (S10 Fig). These important and highly novel findings highlight the importance of the ADAMTS5 enzyme-versican substrate interaction as a key process in the regulation of virus-specific immunity.

## Discussion

Increasing evidence in the literature highlights the importance of zinc-dependent metzincins in the regulation of immune responses. MMPs and ADAMs have been strongly associated with neutrophil, macrophage, dendritic cell, and lymphocyte migration [6,34–36]. Here, we show for the first time that ADAMTS5, a member of the ADAMTS family, has a key role in influenza virus-specific immunity through a mechanism that involves ECM remodelling. *Adamts5*^*-/-*^ mice had higher peak viremias and showed signs of delayed influenza virus clearance when compared to C57.BL/6 controls (Fig 2). The defect contributed to fewer total CD4^+^ and CD8^+^ T cells in the periphery and an accumulation of these cells in the MLN (Figs 3–5). Results from our transwell migration assays and *Adamts5*^*-/-*^*Vcan*^*+/hdf*^ mouse studies further support our hypothesis that the absence of ADAMTS5 reduces ECM proteoglycan cleavage and impedes (but does not entirely block) the movement of influenza-specific lymphocytes to effector sites, such as the lung or to the periphery (Figs 7 and 8).

Migration of CD8^+^ T cells from draining lymph nodes to the periphery is critically important for the establishment of full effector function and eventual clearance of pathogens, such as influenza virus. Our research suggests that the lack of ADAMTS5 enzymatic activity in influenza virus-infected *Adamts5*^*-/-*^ mice results in accumulation of the large extracellular proteoglycan V0/V1 versican (Fig 6A). Increased V0/V1 versican expression has also been noted in the developing limb [11] and heart valves [17] of *Adamts5*^*-/-*^ mice. We believe that the accumulation in the MLN shown in this current study prevents lymphocyte trafficking and results in the exacerbation of disease following influenza virus infection. Furthermore, corroborating evidence by others in the field demonstrates that an epitope in the N-terminal globular domain of versican promoted CD4^+^ T cell migration and lymphocyte rolling [22]. Additionally, versican overexpression was associated with decreased infiltration of CD8^+^ T cells in stromal compartments of cervical cancer [28].

Further studies have suggested that the related zinc-dependent metzincins, the MMPs, are essential for immune cell trafficking. Like ADAMTs enzymes, MMPs contain a catalytic domain that utilises a conserved zinc binding sequence (HEXXHXXXGXX) for catalysing reactions [8] and have a broad range of cleavage substrates. This is in contrast to the highly specific cleavage moieties associated with ADAMTS enzyme activity. It is therefore not surprising that the MMPs have been identified in a vast number of physiological processes [37]. For example, MMP9, a highly characterised extracellular metalloproteinase associated with immune cell trafficking, has been detected in neutrophils, macrophages, dendritic cells, and T cells [31,38]. MMP9 and related MMPs (MMP2, 7, 10, 14) have been shown to degrade ECM roadblocks associated with immune cell trafficking in a similar fashion to what we have proposed in our study. Specifically, MMP9 and MMP2 expressing Th1 T cells demonstrate increased motility through collagen in a transwell migration assay [39]. Supporting in vivo data has suggested that a blockade of the MMP9 and MMP2 signalling pathway (Wnt) is associated with impaired T cell extravasation in an experimentally induced skin inflammation model [6]. Moreover, lipopolysaccharide-stimulated macrophages isolated from *Mmp10*^*-/-*^ mice fail to migrate efficiently in transwell studies when compared to C57.BL/6 control macrophages [5]. In these studies, ECM components, such as collagen and elastin, inhibited immune cell migration. Collagen and elastin form key ECM components of basement membranes, and so dampened MMP activity would, in turn, lead to accumulation of these components and inhibition of immune cell migration and tissue infiltration. In contrast to the abovementioned studies, versican, a key ADAMTS5 substrate, is widely expressed in tissues and is not predominately associated with the basement membrane (as are MMP substrates).

ECM components, such as versican, provide a “sticky” surface for T cell adherence. Versican GAG chains interact directly or indirectly with molecules on the T cell surface, such as CD62L and CD44 [20,21,40], both of which are known to contribute to T cell trafficking. Increased levels of versican, such as those observed in *Adamts5*^*-/-*^ mice, may therefore prevent T cell interaction with the ECM, leading to perturbations in T cell migration. Thus, we propose that cleavage and removal of versican blockades via the action of proteoglycanases, such as ADAMTS5, is required for efficient T cell interaction with the ECM to encourage migration to effector sites in the periphery and for the subsequent resolution of infection (Fig 8G). Our hypothesis is further strengthened by data demonstrating that reduction of versican restores normal T cell function in *Adamts5*^*-/-*^*Vcan*^*+/hdf*^ mice (Fig 8). It is important to note that the migration of influenza-specific CD8^+^ T cells was not fully impeded in our experimental model. ADAMTS5 may therefore be working in concert with other metalloproteinases to facilitate T cell migration. The proteoglycanases, ADAMTS1, 4, 8, 9, 15, and 20, as well as MMP1, 2, 3, 7, and 9, are capable of producing versican fragments in a similar fashion to ADAMTS5 [9,16,32,41,42]. It is reasonable to suspect that there is redundancy built into the trafficking system, as related family members, such as ADAMTS9 (Fig 1F), may be providing some compensatory function in the absence of ADAMTS5, allowing some T cell migration (although highly restricted) to occur into the periphery (Figs 3 and 4). The cooperative requirement of versican cleavage by ADAMTS9 with ADAMTS5 has been observed in embryogenesis, and so its presence in regulation of immune cell migration cannot be discounted [11,43–45]. Furthermore, the transwell migration assay indicated that multiple ADAMTS enzymes can mediate T cell migration (Fig 7A). Further studies with related family members are required to ascertain their specific contribution to influenza-specific immunity.

Our findings would suggest that overexpression of ADAMTS5 or reduced versican expression could restore and improve immunity. Evidence from MMP9-related influenza studies suggests that a more circumspect approach may be required. MMP9 has been shown to be involved in the repair of lung tissue following influenza virus infection and can prevent bleomycin-mediated lung fibrosis by remodelling the ECM and degrading cytokines [46]. However, MMP9 overactivity in MMP9 transgenic mice has been associated with excessive neutrophil infiltration following influenza virus infection, leading to poor survival [38]. An inhibitor targeting ADAMTS5 has already undergone clinical trial as an osteoarthritic therapeutic (https://clinicaltrials.gov/show/NCT00454298). Administration of ADAMTS5 inhibitors for osteoarthritis may therefore be contraindicated in elderly patients, as they are more susceptible to influenza infection. Careful dissection and characterisation of metalloproteinase expression may therefore be required to determine the contribution of these enzymes to overall tissue repair and immunity.

In summary, our data show that the ADAMTS5 ECM enzyme activity is critically important for lymphocyte trafficking following influenza virus infection (especially CD8^+^ T cell immunity). In conclusion, interventions that facilitate increased ADAMTS5 expression used in conjunction with current approved antivirals and/or vaccines offer a new approach for combating unexpected emerging influenza virus pandemic threats.

## Materials and Methods

### Ethics Statement

All animal experiments were approved by the Deakin University Animal Ethics Committee (under G38-2013 and G34-2015) and were conducted in compliance with the guidelines of the National Health and Medical Research Council (NHMRC) of Australia on the care and use of animals for scientific purposes.

### Mice

Six-to-twelve-week old *Adamts5* (B6.129P2-Adamts5^tm1Dgen/J^) and *Vcan* (*Vcan*^*+/hdf*^) male and female mice (Jackson Laboratory), backcrossed eight times on a C57.BL/6 background, were bred at the School of Medicine, Deakin University [11,47]. *Adamts5*^*-/-*^ mice and *Vcan*^*+/hdf*^ mice were crossed for three generations to generate *Adamts5*^*-/-*^*Vcan*^*+/hdf*^ and *Adamts5*^*-/-*^*Vcan*^*+/+*^ mice. The animals were housed at 20°C on a 12-h day/night cycle in sterilised cages (Techniplast) and provided food and water *ad libitum*. Mice were sex and age matched for experiments. C57.BL/6 (WT; wildtype) mice were purchased from the Animal Resource Centre, Perth, Australia.

### Virus Infection

Eight–to-ten-week old male or female naïve C57.BL/6, *Adamts5*^*-/-*^, *Adamts5*^*-/-*^*Vcan*^*+/hdf*^ and *Vcan*^*hdf/+*^ mice were anaesthetized by isoflurane inhalation and infected intranasally (i.n.) with 10^4^ plaque forming units (pfu) X-31 (H3N2) in a 30 μl volume, diluted in PBS. All mice were weighed throughout the course of infection and euthanized at days 3, 7 or 10p.i. We also detail experiments using *Adamts5*^*-/-*^ and WT littermate controls in S11 Fig.

### Tissue Sampling and Cell Preparation

Spleen, lung, and MLN samples were aseptically removed from mice at various time-points following influenza virus infection. Lungs were digested with collagenase (Sigma), whilst spleens and MLNs were disrupted with glass microscope slides to generate single-cell suspensions. Spleen cell suspensions were enriched for T cells following B cell panning on plates coated with goat anti-mouse IgG and IgM antibody (Jackson ImmunoResearch, West Grove, PA) for 1 h at 37°C.

### Viral Titres

Lungs from influenza virus-infected mice were removed and homogenised in RPMI medium 1640 (Life Technologies), containing 40 μg/ml gentamycin and 10,000 μg/ml penicillin/streptomycin. Viral titres (pfu/ml) were determined by plaque assay on Madin-Darby Canine Kidney (MDCK) cell monolayers, as previously described [48].

### Phenotyping Immune Cell Subsets

Spleen, lung, and MLN single cell suspensions from naïve and infected mice were stained with conjugated monoclonal antibodies targeting murine CD3, CD8, CD4, CD314, CD11c, CD11b, MHCII, F4/80, and B220 for 30 min at 4°C and analysed on a BD-LSRII (BD-USA). The following antibodies were purchased from BD Pharmingen: CD8α-PERCP and CD8α-FITC (53–6.7), CD4α-FITC (RM4-4), CD3-APC (145-2C11), F4.80-PE (T45-2342) CD314-PE (CX5), CD11c-APC (HL3), MHCII-PERCP (M5/114), CD11b-FITC (M1/70), and CD45r-PERCP (RAB3-6B2). Results were analysed using Flowjo software version 7 (Flowjo; Ashland, USA).

### Tetramer and Intracellular Cytokine Staining

CD8^+^ T lymphocyte populations from the spleen, lung, and MLN were enumerated following staining with fluorescently labelled tetrameric complexes directed against the two immunodominant influenza-specific CD8^+^ T cells epitopes (D^b^NP_366-372_-PE or D^b^PA_224-233_-PE) for 1 h at room temperatures in 0.1% BSA/ 0.02% sodium azide in PBS, as previously described [49,50]. Cells were then washed and stained with anti-CD8α-FITC and analysed on a BD-LSRII. D^b^NP_366-372_ and D^b^PA_224-233_ CD8^+^ T function was then assessed using ICS. Briefly, cells were cultured for 5 h in 96-well round bottom plates with influenza NP_366-372_ (ASNENMETM) or PA_224-233_ (SSLENFRAYV) peptide in the presence of Golgi-plug (BD, USA) and IL-2, permeabilised and stained for the presence of CD8α and IFNγ (BD, USA) as previously described [51]. Data was acquired on a BD-LSRII and analysed using Flowjo software.

### ADAMTS and Influenza Virus Gene Expression Levels

RNA was extracted from CD8^+^ T cells in the lung and spleen of C57.BL/6 and *Adamts5*^*-/-*^ mice, immortalised CD4^+^ T cells (JURKAT cells), and human peripheral blood lymphocytes as per the manufacturer’s instructions using an RNeasy kit (Qiagen). One microgram of total RNA was reverse transcribed using the Superscript III cDNA synthesis kit (LifeTech). QRT-PCR was undertaken on cDNA using iQ SYBR Green Super mix (Bio-Rad) and oligonucleotide primers for ADAMTS proteoglycanases 1, 4, 5, 8, 9, 15, and 20 with the following qRT-PCR parameters: 94°C for 2 min followed by 40 cycles of 94°C for 15 s and 58°C for 1 min. Influenza M1 cDNA levels were measured using probes as previously described (Life Technologies) [52]. The quant-iT OliGreen ssDNA Assay Kit (Invitrogen) was used to quantitate total cDNA input following manufacturer’s instructions. Changes in mRNA levels in lungs were calculated using 2^-ΔΔ*Ct*^ method [16].

### Construct Expression in HEK293T Cells

Human embryonic kidney (HEK) 293T cells (ATCC, Manassas, VA) were grown in DMEM (Gibco) containing 10% FCS in 5% CO_2_ at 37°C. Cells were transfected using Lipofectamine 2000 (Invitrogen) with pcDNA3.1MycHisA+ (Invitrogen) constructs encoding mouse full length V1 versican (kindly provided by Professor Dieter Zimmerman), full-length ADAMTS5, catalytically inactive ADAMTS5 (ADAMTS5EA), full-length ADAMTS15 and catalytically inactive ADAMTS15 (ADAMTS15EA), and empty vector control according to the manufacturer’s instructions. Serum-free conditioned medium was collected at 48 h post transfection as previously described, and expression was detected by western blotting using an anti-GAG antibody (Merck Millipore) and anti-myc (Merck Millipore) antibody for transfection with ADAMTS constructs.

### Versican Cleavage Assays

CD8^+^ T cells from influenza virus infected *Adamts5*^*-/-*^ and C57.BL/6 mice were incubated with HEK293T conditioned media containing full-length V1 versican and IL2 (20 U/mL) for 16 h at 37°C. Cleavage was detected by western blotting using an anti-GAG (versican) antibody (Merck Millipore) and anti-DPEAAE (versikine) antibody (Abcam). Immunoblots were analysed using ImageJ software.

### Ex Vivo Migration Assays

Migration assays were performed in 12-well chamber inserts (5 μM) (Corning Inc.) as previously described [39]. Inserts were coated with V1 versican conditioned media [16,32], and recombinant mouse CXCL12 (10ng/ml) (R&D) was added to the lower chamber of the transwell to promote migration. *Adamts5*^*-/-*^ or C57.BL/6 magnetically-enriched (Stemcell) CD8^+^ T spleen cells (10^5^) were loaded to the upper chamber in migration media containing ADAMTS5FL, ADAMTS5EA, ADAMTS15FL, or ADAMTS15EA conditioned media from tramsfected HEK293T cells or serum-free migration media. Additionally, ADAMTS5 antibody (1000 ng/mL) was added to the upper chamber of JURKAT cells (10^5^) in the versican transwell chamber and migration assessed. These cells were allowed to migrate for 4 h at 37°C at 5% CO_2._ Following removal of non-migrating cells in the upper chamber, the transwell membrane was stained with haematoxylin (Sigma-Aldrich) to determine the number of migrating cells.

### B-Galactosidase Staining

MLNs from infected C57.BL/6 and *Adamts5*^*-/-*^ mice were removed at day 10p.i. and fixed in 4% paraformaldehyde prepared in β-gal wash buffer (0.1 M phosphate buffer pH 7.4, 2 mM MgCl_2_, 0.02% NP-40, 0. 01% Na deoxycholate). MLNs were then washed in this buffer and incubated overnight in β-gal staining solution (5 mM potassium ferricyanide, 5 mM potassium ferrocyanide, 1 mg/ml X-gal in DMSO) at 37°C. The next day, MLNs were rinsed in wash buffer and fixed in 4% paraformaldehyde (prepared in wash buffer) at 4°C overnight. After a brief rinse in wash buffer, tissues were imaged then embedded in paraffin for sectioning and eosin staining.

### Immunohistochemistry

MLNs from infected C57.BL/6 and *Adamts5*^*-/-*^ mice were fixed in 4% paraformaldehyde at 4°C overnight and then paraffin-embedded and sectioned. Seven micrometre sections were stained with anti-versican (Merck-Millipore) or anti-DPEEAE (versican cleavage fragment) (Thermo-Fischer) antibodies at 4°C overnight. The following day, tissues were washed in 10% Triton-X (Astral) to remove excess antibody and then incubated with Alexa-fluor594 goat-anti-mouse antibody (Life-technologies). Sections were then washed in 10% X Triton-X (3 x 10 min) and stained with DAPI (Thermo-Fischer). Slides were then viewed under a Leica SP5 confocal microscope at 400 x magnification.

### Statistics

As data were normally distributed, they are presented as grouped data expressed as mean ± standard deviation (SD); *n* represents the number of mice. Statistical differences between two groups were analysed by Student's *t* test. Statistical differences between more than two groups were determined by two-way analysis of variance (ANOVA), followed by a Bonferroni multiple-comparison test. All statistical analyses were performed using GraphPad Prism 5 for Windows. In all cases, probability levels less than 0.05 (*p < 0.05) were indicative of statistical significance.

## Supporting Information

S1 Data Underlying data for Figs 1–8. (XLSX)

S2 Data Underlying data for S1–S4 Figs and S7–S10 Figs. (XLSX)

S1 Fig Immune cell subsets in the lungs of naïve *Adamts5*^*-/-*^ mice. Lungs from naïve C57.BL/6 and *Adamts5*^*-/-*^ mice were removed and immune cell subsets were characterised. **(A)** Dendritic cells (CD11c^+^MHCII^+^), **(B)** macrophages (CD11b^+^F4/80^+^), **(C)** NK cells (CD314^+^CD3^-^), **(D)** CD8^+^ T cells, **(E)** CD4^+^ T, cells and **(F)** B cells (B220^+^) in the lung of naïve C57.BL/6 and *Adamts5*^*-/-*^ mice. WT denotes C57.BL/6 mice. Results are expressed as means ± SD, and statistical significance (*p* < 0.05 relative to C57.BL/6) determined by Student’s *t* test (*n* = 5 mice representing three experiments). Underlying data are provided in S2 Data.
(TIF)

S2 Fig Immune cell subsets in the spleen of naïve *Adamts5*^*-/-*^ mice. Spleen from naïve C57.BL/6 and *Adamts5*^*-/-*^ mice were removed and immune cell subsets were characterised. **(A)** Dendritic cells (CD11c^+^MHCII^+^), **(B)** macrophages (CD11b^+^F4/80^+^), **(C)** NK cells (CD314^+^CD3^-^), **(D)** CD8^+^ T cells, **(E)** CD4^+^ T cells, and **(F)** B cells (B220^+^) in the spleen of naïve C57.BL/6 and *Adamts5*^*-/-*^ mice. WT denotes C57.BL/6 mice. Results are expressed as means ± SD, and statistical significance (*p* < 0.05 relative to C57.BL/6) determined by Student’s *t* test (*n* = 5 mice representing three experiments). Underlying data are provided in S2 Data.
(TIF)

S3 Fig T cell subsets in the thymus of naïve *Adamts5*^*-/-*^ mice. Thymus from naïve C57.BL/6 and *Adamts5*^*-/-*^ mice were removed and immune cell subsets were characterised. Total **(A)** CD4^+^ and **(B)** CD8^+^ T cells in the thymus of naïve C57.BL/6 and *Adamts5*^*-/-*^ mice. WT denotes C57.BL/6 mice. Results are expressed as means ± SD, and statistical significance (*p* < 0.05 relative to C57.BL/6) determined by Student’s *t* test (*n* = 5 mice representing three experiments). Underlying data are provided in S2 Data.
(TIF)

S4 Fig ADAMTS expression levels in the lungs of *Adamts5*^*-/-*^ mice. cDNA from the lungs of influenza virus infected *Adamts5*^*-/-*^ and C57.BL/6 mice was generated and the expression of ADAMTS enzymes assessed by qRT-PCR. Expression of ADAMTS **(A)** 1, **(B)** 4, **(C)** 5, **(D)** 8, **(E)** 9, and **(F)** 15 enzymes at 0, 3, 7, and 10 d p.i. WT denotes C57.BL/6 mice. Results are expressed as means ± SD, and statistical significance (*p* < 0.05 relative to C57.BL/6 controls) determined by Student’s *t* test (*n* = 5 mice representing three experiments). Underlying data are provided in S2 Data.
(TIF)

S5 Fig T cells colocalize with versican. C57.BL/6 and *Adamts5*^*-/-*^ mice were infected i.n. (10^4^ pfu/mouse) with X31 (H3N2) influenza virus. MLNs were removed, sectioned, and stained for expression of versican (GAGβ) and CD3 (T cells). **(A)** Versican and T cell staining in the MLN of C57.BL/6 and *Adamts5*^*-/-*^ mice was assessed day 7 p.i. Blue = DAPI, Red = versican (GAGβ), Green = CD3. **(B)** qRT-PCR of versican in the MLN. (*n* = 3 representing three separate experiments). Underlying data are provided in S2 Data.
(TIFF)

S6 Fig Versican and versikine expression in the lungs of *Adamts5*^*-/-*^ mice. Sections of lungs from influenza virus infected *Adamts5*^*-/-*^ and C57.BL/6 mice were assessed for the expression of versican and versikine by immunofluorescence. **(A)** Versican expression in the bronchiole and **(B)** versikine in the artery of the lung. (*n* = 15). WT denotes C57.BL/6 mice.
(TIF)

S7 Fig ADAMTS enzyme expression and the role of ADAMTS5 in human T cell migration. cDNA from immortalised CD4^+^ T cells (JURKAT cells) and peripheral blood lymphocytes was assessed for the expression of ADAMTS enzymes by qRT-PCR. Expression of ADAMTS 1, 4, 5, 8, 9, 15, and 20 enzymes in **(A)** JURKAT cells and **(B)** peripheral blood lymphocytes. **(C)** JURKAT cells were treated with an ADAMTS5 antibody, and migration through a versican overlay is shown by graphical representation. WT denotes C57.BL/6 mice. Results are expressed as means ± SD, and statistical significance (*p* < 0.05 relative to C57.BL/6 controls) determined by Student’s *t* test (*n* = 5 mice representing three experiments). Underlying data are provided in S2 Data.
(TIF)

S8 Fig ADAMTS enzymes expressed by CD8^+^ T cells. CD8^+^ T cells from the spleen of influenza virus infected C57.BL/6 and *Adamts5*^*-/-*^ mice were assessed for the expression of ADAMTS enzymes (ADAMTS1, 4, 5, 9, and 15) using qRT-PCR. WT denotes C57.BL/6 mice. Results are expressed as means ± SD, and statistical significance (*p* < 0.05 relative to C57.BL/6 controls) determined by Student’s *t* test (*n* = 5 mice representing three experiments). Underlying data are provided in S2 Data.
(TIF)

S9 Fig Cleavage of versican by CD8^+^ T cells. CD8^+^ T cells were isolated from influenza virus infected *Adamts5*^*-/-*^ and C57.BL/6 mice and incubated with versican-conditioned media for 16 hours. Versican cleavage is shown by **(A)** western blot analysis and **(B)** densitometric quantification of protein bands using Image J software. Results are expressed as means ± SD, and statistical significance (*p* < 0.05 and *p* < 0.005 relative to C57.BL/6 controls) determined by Student’s *t* test (*n* = 5 representing three experiments). Underlying data are provided in S2 Data.
(TIF)

S10 Fig Influenza virus infection of *Vcan*^*+/hdf*^ mice. Lung tissue and MLNs were removed from influenza virus infection C57.BL/6 and *Vcan*^*+/hdf*^ mice and processed to generate single cell suspensions at day 10 p.i. for analysis of influenza-specific immunity. **(A)** Total CD8^+^ T cell numbers were determined at day 10 p.i. in the lung. **(B)** Influenza-specific D^b^NP_366-372_^+^ CD8^+^ and D^b^PA_224-233_^+^ CD8^+^ tetramer positive T cells in the lung were enumerated at day 10 p.i. CD8^+^ T cell functionality was measured using ICS. **(C)** Influenza specific D^b^NP_366-372_^+^IFNγ^+^CD8^+^ and D^b^PA_224-233_^+^IFNγ^+^CD8^+^ T cell responses were characterised in the lung at day 10 p.i. **(D)** Total CD8^+^ T cell numbers were determined at day 10 p.i. from pooled MLN samples. **(E)** Influenza-specific D^b^NP_366-372_^+^ CD8^+^ and D^b^PA_224-233_^+^ CD8^+^ tetramer positive T cells in pooled MLN were enumerated at day 10 p.i. **(F)** CD8^+^ T cell functionality was measured using ICS to assess influenza-specific D^b^NP_366-372_^+^IFNγ^+^CD8^+^ and D^b^PA_224-233_^+^IFNγ^+^CD8^+^ T cell responses at day 10 p.i. The results are expressed as means ± SD or as pooled means (MLN data) and statistical significance (relative to C57.BL/6 mice) determined by a Student’s *t* test (*p ≤ 0.05, ***p ≤ 0.005 relative to C57.BL/6, *n* = 5 representing three individual experiments). WT denotes C57.BL/6 mice. Underlying data are provided in S2 Data.
(TIFF)

S11 Fig Influenza infection of *Adamts5*^*-/-*^ and WT littermate controls. *Adamts5*^*-/-*^ and WT mice were infected i.n with X31 (H3N2) influenza virus and spleens, lungs, and MLNs removed from C57.BL/6 and *Adamts5*^*-/-*^ mice days 7 p.i. Single cell suspensions were then analysed for influenza-specific immunity. **(A)** Weight loss was calculated over the time course of infection. Total CD4^+^ and CD8^+^ T cells were enumerated in the **(B)** spleen, **(C)** lung, and **(D)** MLN. Influenza-specific D^b^NP_366-372_^+^ CD8^+^ and D^b^PA_224-233_^+^ CD8^+^ tetramer positive T cell numbers were also characterised in the (**B)** spleen, **(C)** lung, and **(D)** MLN. Lung and spleen results are expressed as means ± SD or as pooled means (MLN data), and statistical significance (relative to C57.BL/6 mice) was determined by a Student’s *t* test (*p ≤ 0.05, **p ≤ 0.01 relative to C57.BL/6 mice, *n* = 5 representing three individual experiments). WT denotes C57.BL/6 mice. Underlying data are provided in S2 Data.
(TIF)

## Abbreviations

ADAMTS: A Disintegrin-like and Metalloproteinase with Thrombospondin-1 motifs
CSPG: chondroitin sulphate proteoglycan
ECM: extracellular matrix
GAG: glycosaminoglycan
ICS: intracellular cytokine staining
i.n.: intranasally
MLN: mediastinal lymph node
MMP: matrix metalloproteinase
p.i.: post infection
QRT-PCR: quantitative reverse-transcriptase polymerase chain reaction
WT: wild-type
