# Correction: ADAMTS5 Is a Critical Regulator of Virus-Specific T Cell Immunity

**DOI:** 10.1371/journal.pbio.3000558

**Published:** 2019-11-06

**Authors:** Meagan McMahon, Siying Ye, Leonard Izzard, Daniel Dlugolenski, Ralph A. Tripp, Andrew G. D. Bean, Daniel R. McCulloch, John Stambas

An influenza-specific CD8+ T cell peptide encoded within the nucleoprotein (NP) was incorrectly referred to as NP366-372 throughout the article. The correct designation should be NP366-374. The peptide sequence ASNENMETM stated throughout the ‘Methods’ section should also be attributed to NP366-374.

The authors have provided corrected versions of Fig 3, Fig 4, Fig 5, Fig 8, S10 Fig and S11 Fig.

**Fig 3:**
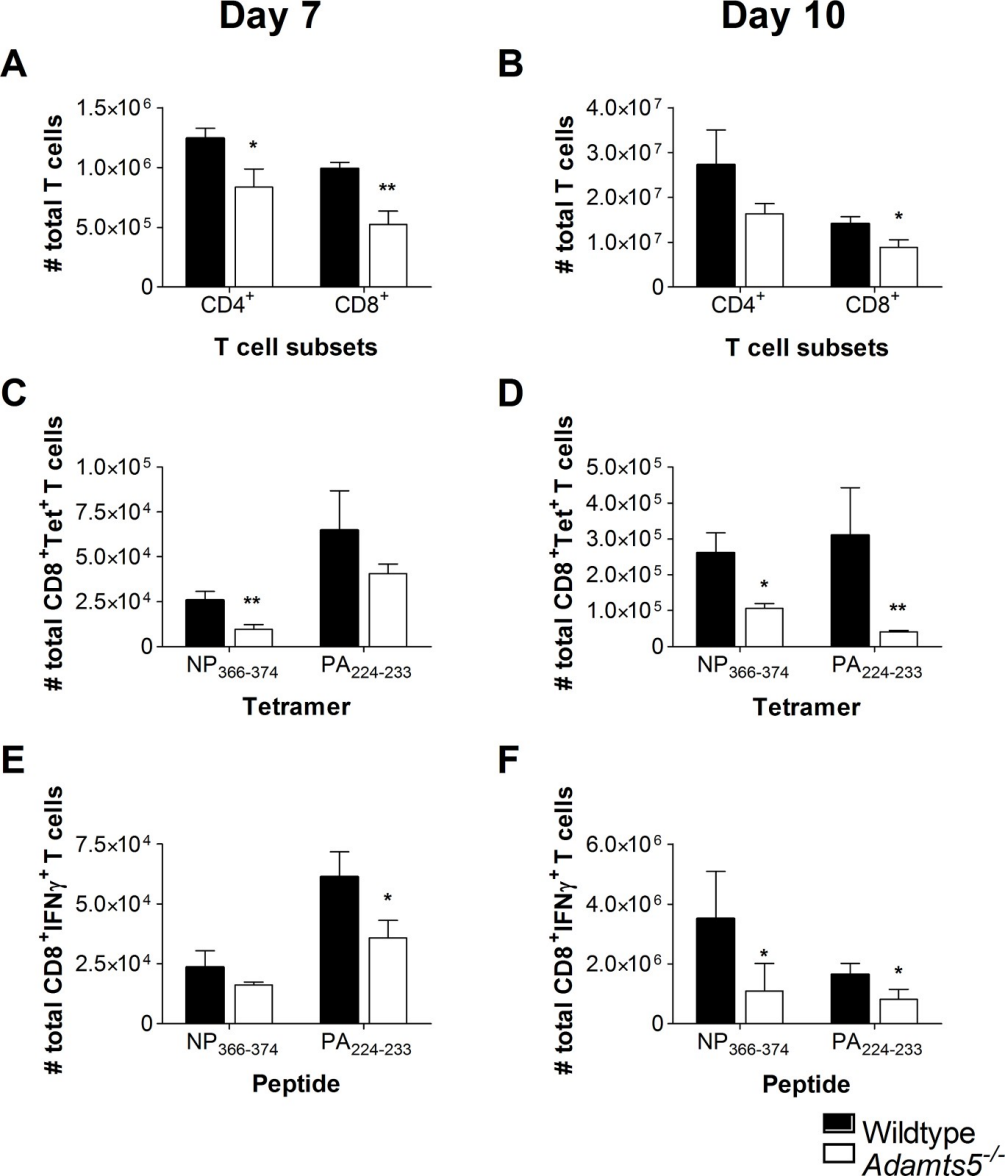
Reduced CD4^+^ and CD8^+^ T cell numbers in the spleens of influenza-infected *Adamts5*^*-/-*^ mice. C57.BL/6 and *Adamts5*^*-/-*^ mice were intranasally (i.n.) infected (10^4^ pfu/mouse) with X31 (H3N2) influenza virus. Spleens were removed at day 7 and day 10 p.i., and CD8^+^ T cell responses determined. Total CD4^+^ and CD8^+^ T cells numbers were calculated at days **(A)** 7 and **(B)** 10 p.i. Influenza-specific D^b^NP_366-374_ CD8^+^ and D^b^PA_224-233_ CD8^+^ tetramer-positive T cell numbers were measured at days **(C)** 7 and **(D)** 10 p.i. Functional influenza-specific D^b^NP_366-374_^+^IFNγ^+^CD8^+^ and D^b^PA_224-233_^+^IFNγ^+^CD8^+^ T cell activity was determined by ICS, and total IFNγ^+^ T cells enumerated at days **(E)** 7 and **(F)** 10 after infection. WT denotes C57.BL/6. The results are expressed as means ± SD, and statistical significance (relative to C57.BL/6) was determined by Student’s *t* test (*p ≤ 0.05, **p ≤ 0.01, *n* = 5 representing three experiments). Underlying data are provided in S1 Data.

**Fig 4:**
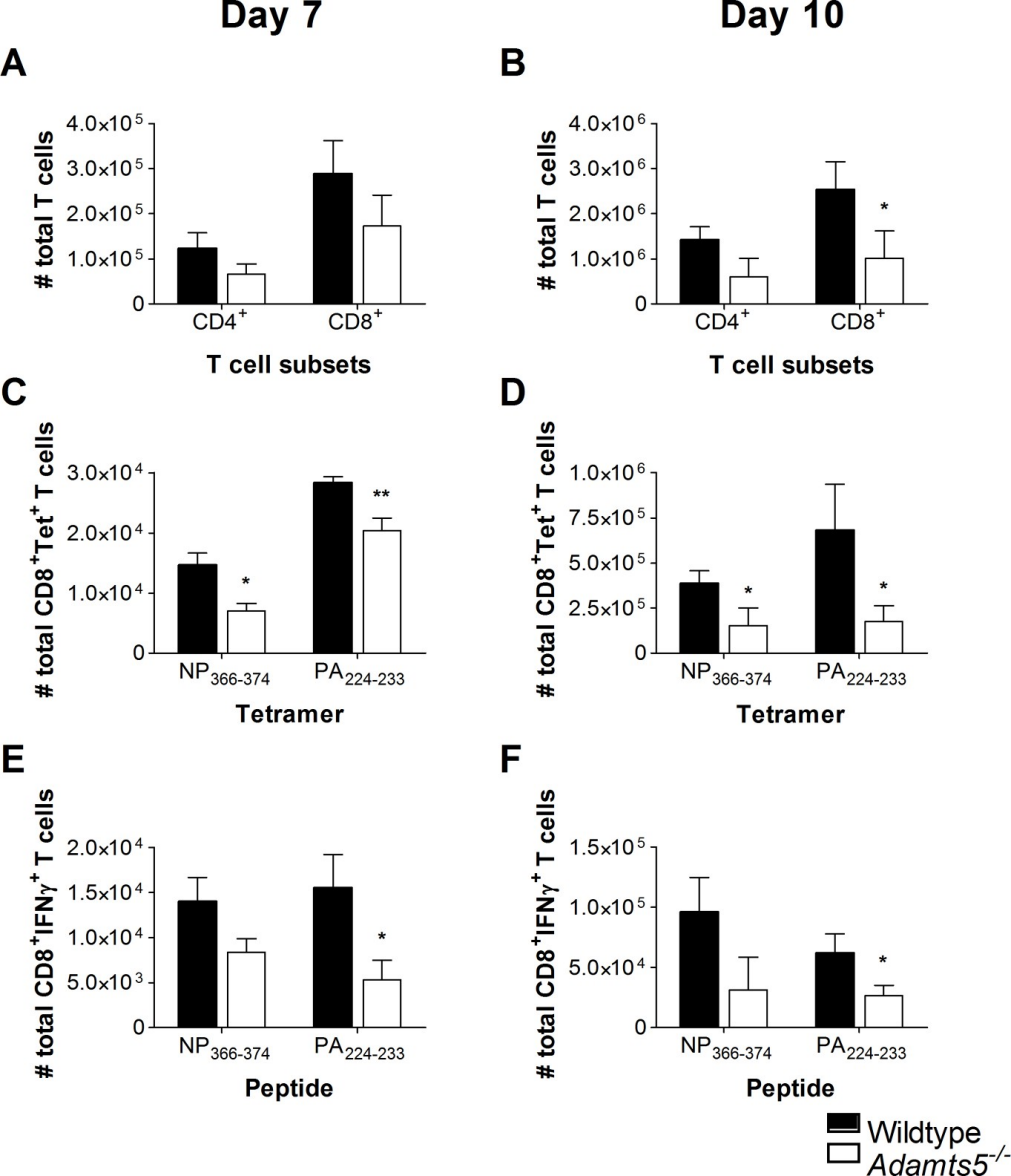
CD4^+^ and CD8^+^ T cell responses in the lungs of influenza-infected *Adamts5*^*-/-*^ mice. C57.BL/6 and *Adamts5*^*-/-*^ mice were infected i.n. with 10^4^pfu X31 (H3N2) influenza virus. Lungs were removed at days 7 and 10 p.i., and CD8^+^ T cell immunity characterised. Total CD4^+^ and CD8^+^ T cell numbers are shown at days **(A)** 7 and **(B)** 10 p.i. Influenza-specific tetramer^+^ D^b^NP_366-374_^+^ CD8^+^ and D^b^PA_224-233_^+^ CD8^+^ T cell responses were enumerated at days **(C)** 7 and **(D)** 10 p.i. CD8^+^ T cell functionality was assessed by ICS and D^b^NP_366-374_^+^IFNγ^+^CD8^+^ and D^b^PA_224-233_^+^IFNγ^+^CD8^+^ T cell responses enumerated at days **(E)** 7 and **(F)** 10 p.i. Wildtype denotes C57.BL/6 mice. The results are expressed as means ± SD, and statistical significance (relative to C57.BL/6) was determined by Student’s *t* test (* = *p* ≤ 0.05, ** = *p ≤* 0.01, *n* = 5 representing three experiments). Underlying data are provided in S1 Data.

**Fig 5:**
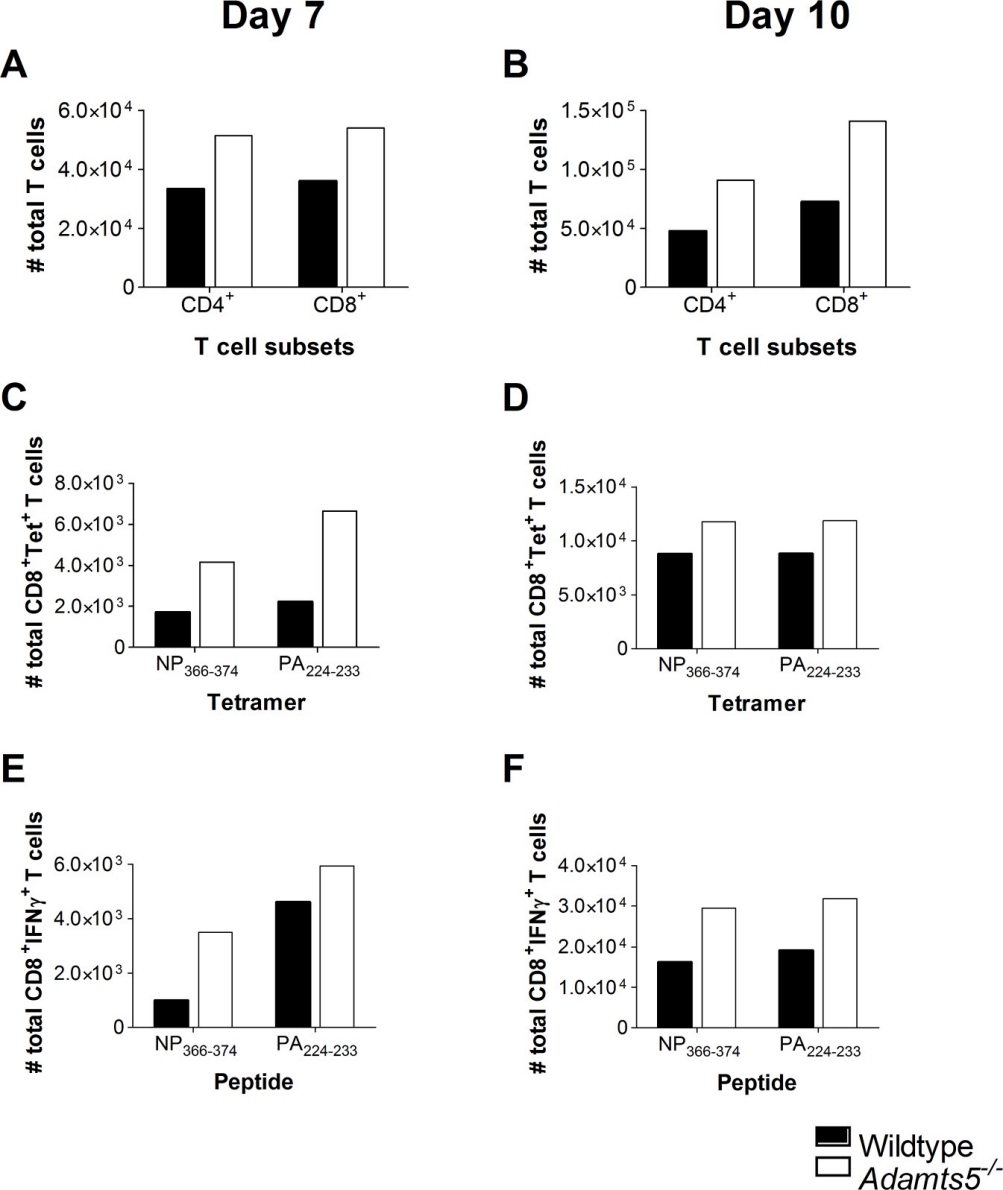
T cell immunity in the pooled MLN. C57.BL/6 and *Adamts5*^*-/-*^ mice were infected i.n. with 10^4^ pfu/mouse X31 (H3N2) influenza virus. MLNs were removed, pooled, and processed at days 7 and 10 p.i., and single-cell suspensions analysed for influenza-specific immunity. Total CD4^+^ and CD8^+^ T cell numbers were determined at days **(A)** 7 and **(B)** 10 p.i. Influenza-specific D^b^NP_366-374_^+^ CD8^+^ and D^b^PA_224-233_^+^ CD8^+^ tetramer positive T cells were enumerated at days **(C)** 7 and **(D)** 10 p.i. CD8^+^ T cell functionality was measured using ICS. Influenza-specific D^b^NP_366-374_^+^IFNγ^+^CD8^+^ and D^b^PA_224-233_^+^IFNγ^+^CD8^+^ T cell responses were characterised at days **(E)** 7 and **(F)** 10 p.i. Results are expressed as total pooled means from five mice repeated three times. Wildtype denotes C57.BL/6 mice. Underlying data are provided in S1 Data.

**Fig 8:**
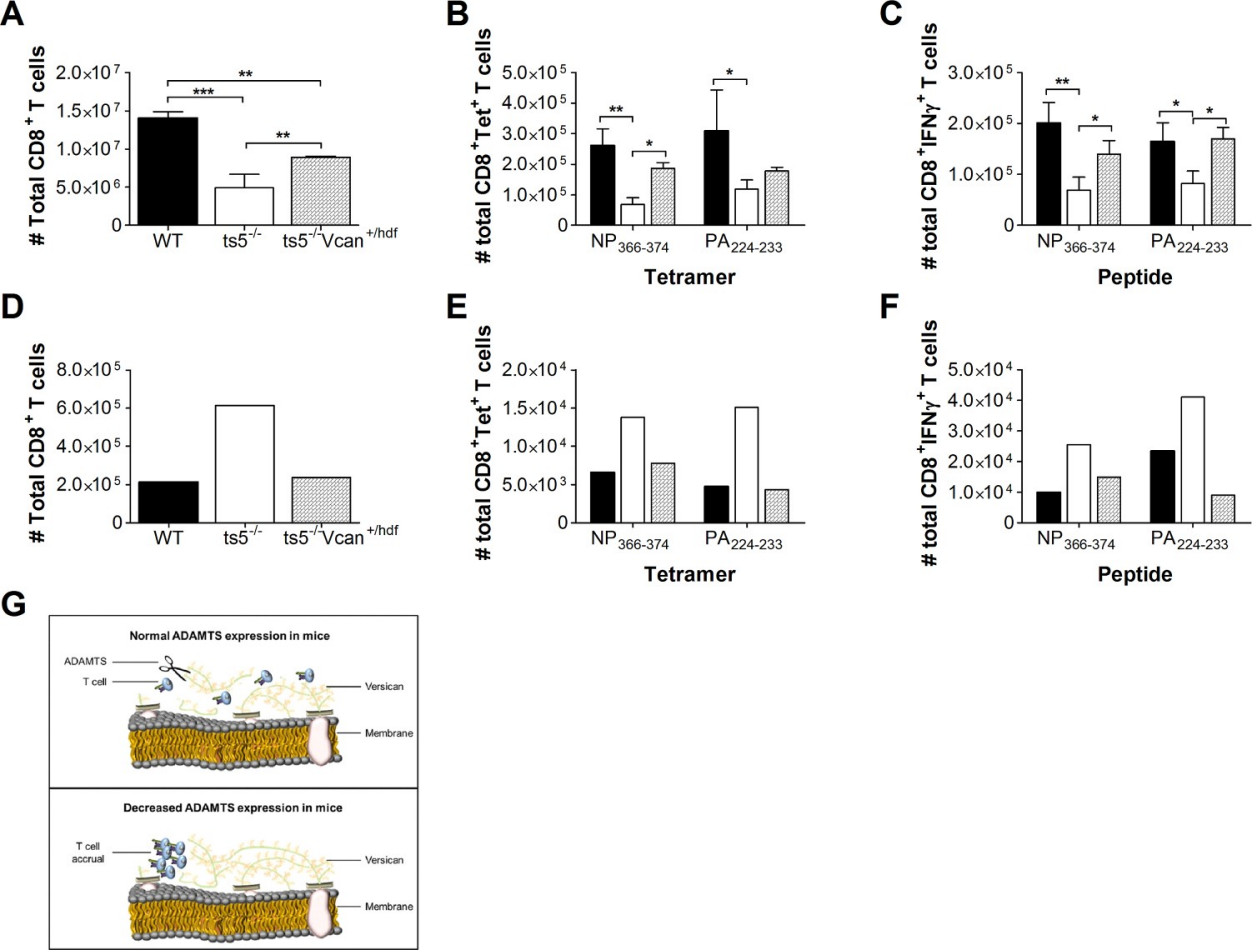
Versican reduction in *Adamts5*^*-/-*^*Vcan*^*+/hdf*^ mice restores normal T cell function. Spleen and MLNs were removed from C57.BL/6, *Adamts5*^*-/-*^*Vcan*^*+/+*^, and *Adamts5*^*-/-*^*Vcan*^*+/hdf*^ mice and processed at day 10 p.i., and single cell suspensions were analysed for influenza-specific immunity. **(A)** Total CD8^+^ T cell numbers were determined at day 10 p.i. in the spleen. **(B)** Influenza-specific D^b^NP_366-374_^+^ CD8^+^ and D^b^PA_224-233_^+^ CD8^+^ tetramer positive T cells in the spleen were enumerated at day 10 p.i. CD8^+^ T cell functionality was measured using ICS. **(C)** Influenza specific D^b^NP_366-374_^+^IFNγ^+^CD8^+^ and D^b^PA_224-233_^+^IFNγ^+^CD8^+^ T cell responses were characterised in the spleen at days 10 p.i. **(D)** Total CD8^+^ T cell numbers were assessed at day 10 p.i. in the pooled MLN. **(E)** Influenza-specific D^b^NP_366-374_^+^ CD8^+^ and D^b^PA_224-233_^+^ CD8^+^ tetramer positive T cells in the pooled MLN were enumerated at day 10 p.i. CD8^+^ T cell functionality was measured using ICS. **(F)** Influenza-specific D^b^NP_366-374_^+^IFNγ^+^CD8^+^ and D^b^PA_224-233_^+^IFNγ^+^CD8^+^ T cell responses were characterised in the pooled MLN at day 10 p.i. The results are expressed as means ± SD (spleen data) or as pooled means (MLN data), and statistical significance (relative to C57.BL/6 mice) was determined by one-way ANOVA (*p ≤ 0.05, ***p ≤ 0.005 relative to C57.BL/6, *n* = 5 representing three individual experiments). WT denotes C57.BL/6 mice and *ts5*^*-/-*^ denotes *Adamts5*^*-/-*^. **(G)** Our model for ADAMTS5 enzyme activity and T cell migration proposes that versican can inhibit T cell effector function by acting as a physical block. Cleavage of versican by ADAMTS5 removes the ECM blockade, allowing migration (top panel). Moreover, versican accumulation in the absence of ADAMTS enzyme activity results in T cell clustering (bottom panel). Underlying data are provided in S1 Data.

The relevant figure legends have been amended to reflect this change and are presented below.

## Supporting information

S10 Fig Influenza virus infection of *Vcan*^*+/hdf*^ mice. Lung tissue and MLNs were removed from influenza virus infection C57.BL/6 and *Vcan*^*+/hdf*^ mice and processed to generate single cell suspensions at day 10 p.i. for analysis of influenza-specific immunity. **(A)** Total CD8^+^ T cell numbers were determined at day 10 p.i. in the lung. **(B)** Influenza-specific D^b^NP_366-374_^+^ CD8^+^ and D^b^PA_224-233_^+^ CD8^+^ tetramer positive T cells in the lung were enumerated at day 10 p.i. CD8^+^ T cell functionality was measured using ICS. **(C)** Influenza specific D^b^NP_366-374_^+^IFNγ^+^CD8^+^ and D^b^PA_224-233_^+^IFNγ^+^CD8^+^ T cell responses were characterised in the lung at day 10 p.i. **(D)** Total CD8^+^ T cell numbers were determined at day 10 p.i. from pooled MLN samples. **(E)** Influenza-specific D^b^NP_366-374_^+^ CD8^+^ and D^b^PA_224-233_^+^ CD8^+^ tetramer positive T cells in pooled MLN were enumerated at day 10 p.i. **(F)** CD8^+^ T cell functionality was measured using ICS to assess influenza-specific D^b^NP_366-374_^+^IFNγ^+^CD8^+^ and D^b^PA_224-233_^+^IFNγ^+^CD8^+^ T cell responses at day 10 p.i. The results are expressed as means ± SD or as pooled means (MLN data) and statistical significance (relative to C57.BL/6 mice) determined by a Student’s *t* test (*p ≤ 0.05, ***p ≤ 0.005 relative to C57.BL/6, *n* = 5 representing three individual experiments). WT denotes C57.BL/6 mice. Underlying data are provided in S2 Data.
(TIF)

S11 Fig Influenza infection of *Adamts5*^*-/-*^ and WT littermate controls. *Adamts5*^*-/-*^ and WT mice were infected i.n with X31 (H3N2) influenza virus and spleens, lungs, and MLNs removed from C57.BL/6 and *Adamts5*^*-/-*^ mice days 7 p.i. Single cell suspensions were then analysed for influenza-specific immunity. **(A)** Weight loss was calculated over the time course of infection. Total CD4^+^ and CD8^+^ T cells were enumerated in the **(B)** spleen, **(C)** lung, and **(D)** MLN. Influenza-specific D^b^NP_366-374_^+^ CD8^+^ and D^b^PA_224-233_^+^ CD8^+^ tetramer positive T cell numbers were also characterised in the (**B)** spleen, **(C)** lung, and **(D)** MLN. Lung and spleen results are expressed as means ± SD or as pooled means (MLN data), and statistical significance (relative to C57.BL/6 mice) was determined by a Student’s *t* test (*p ≤ 0.05, **p ≤ 0.01 relative to C57.BL/6 mice, *n* = 5 representing three individual experiments). WT denotes C57.BL/6 mice. Underlying data are provided in S2 Data.
(TIF)
