# Supplementary figures and images for: ADAMTS5 Is a Critical Regulator of Virus-Specific T Cell Immunity
Source: PLoS Biol. 2016 Nov 17;14(11):e1002580. doi: 10.1371/journal.pbio.1002580 (PMC5113859; doi:10.1371/journal.pbio.1002580)

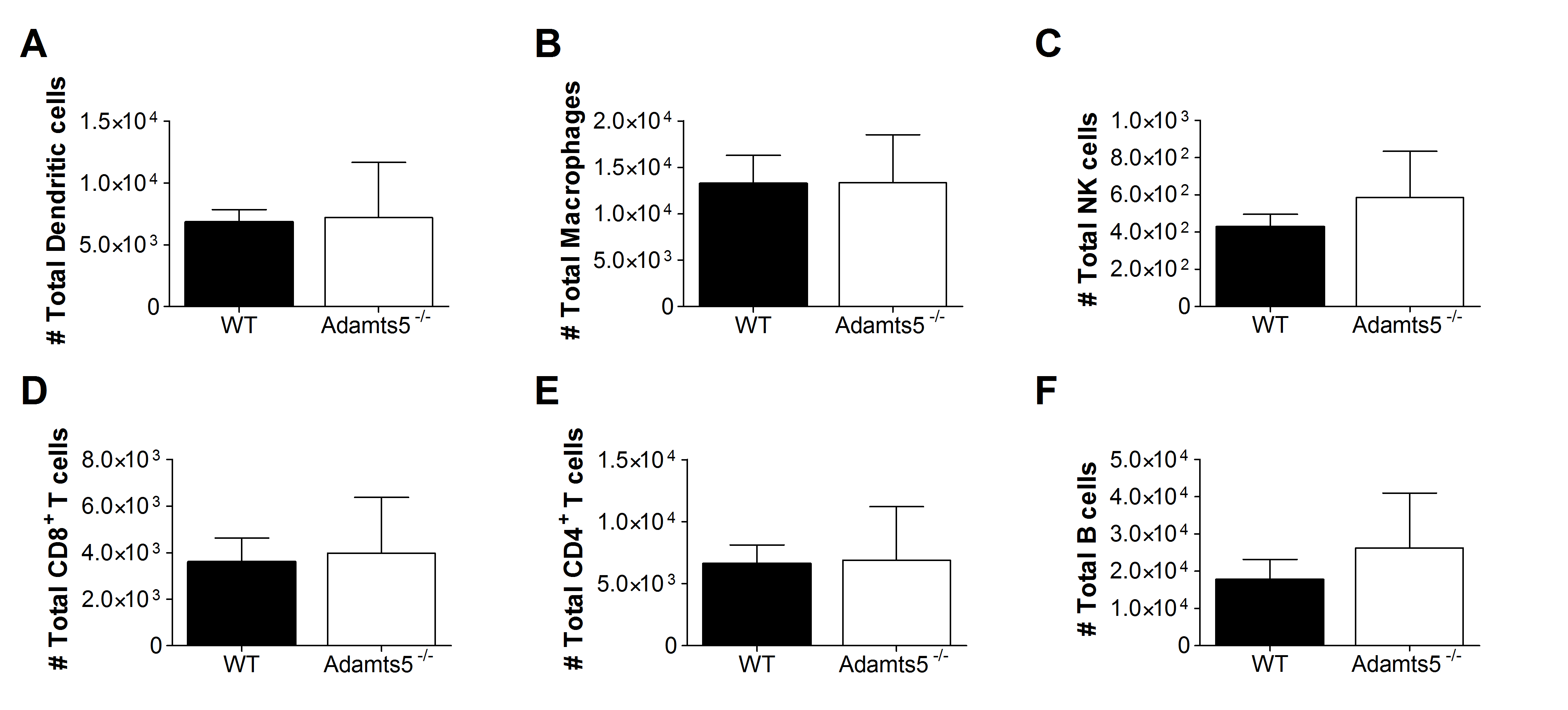

Supplement: S1 Fig — Lungs from naïve C57.BL/6 and Adamts5-/- mice were removed and immune cell subsets were characterised. (A) Dendritic cells (CD11c+MHCII+), (B) macrophages (CD11b+F4/80+), (C) NK cells (CD314+CD3-), (D) CD8+ T cells, (E) CD4+ T, cells and (F) B cells (B220+) in the lung of naïve C57.BL/6 and Adamts5-/- mice. WT denotes C57.BL/6 mice. Results are expressed as means ± SD, and statistical significance (p < 0.05 relative to C57.BL/6) determined by Student’s t test (n = 5 mice representing three experiments). Underlying data are provided in S2 Data. (TIF) [file pbio.1002580.s003.tif]

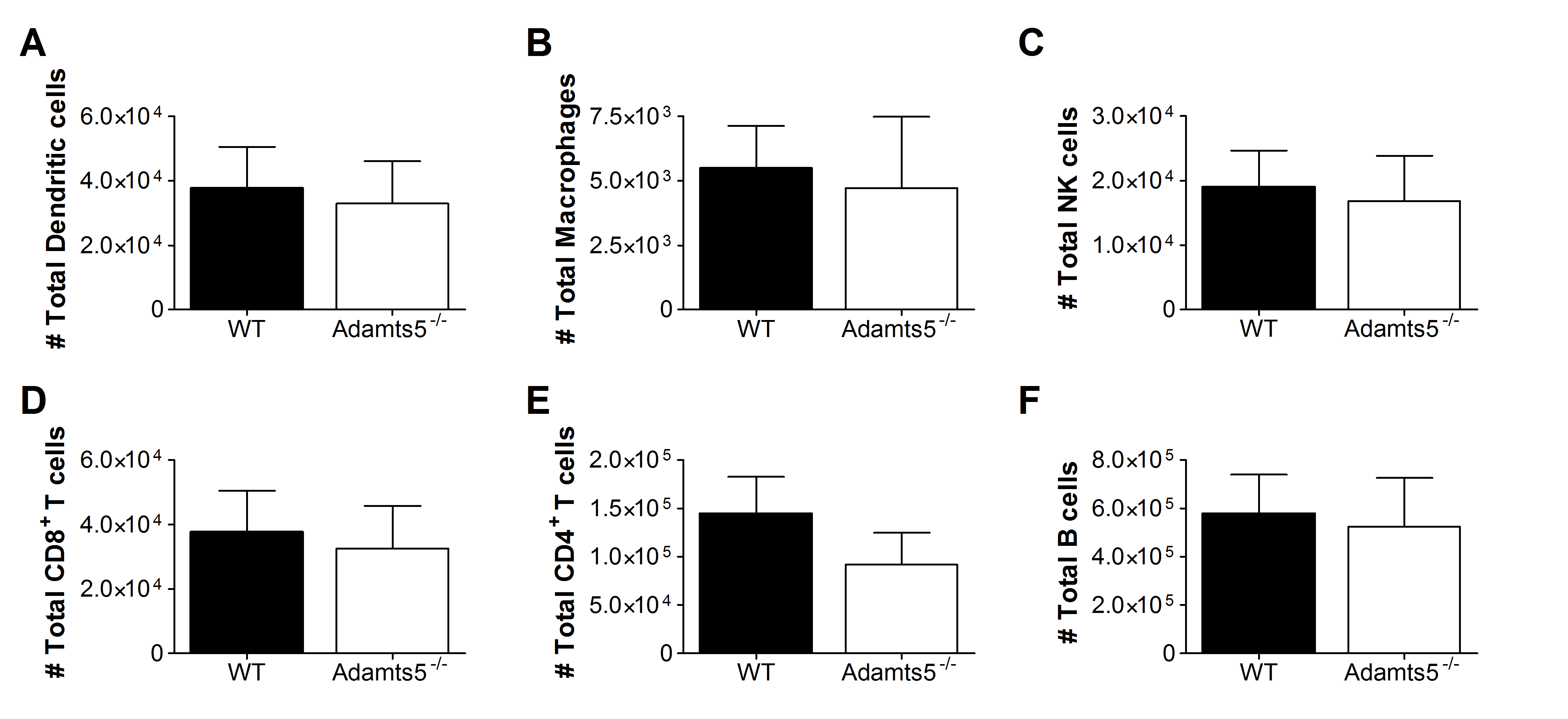

Supplement: S2 Fig — Spleen from naïve C57.BL/6 and Adamts5-/- mice were removed and immune cell subsets were characterised. (A) Dendritic cells (CD11c+MHCII+), (B) macrophages (CD11b+F4/80+), (C) NK cells (CD314+CD3-), (D) CD8+ T cells, (E) CD4+ T cells, and (F) B cells (B220+) in the spleen of naïve C57.BL/6 and Adamts5-/- mice. WT denotes C57.BL/6 mice. Results are expressed as means ± SD, and statistical significance (p < 0.05 relative to C57.BL/6) determined by Student’s t test (n = 5 mice representing three experiments). Underlying data are provided in S2 Data. (TIF) [file pbio.1002580.s004.tif]

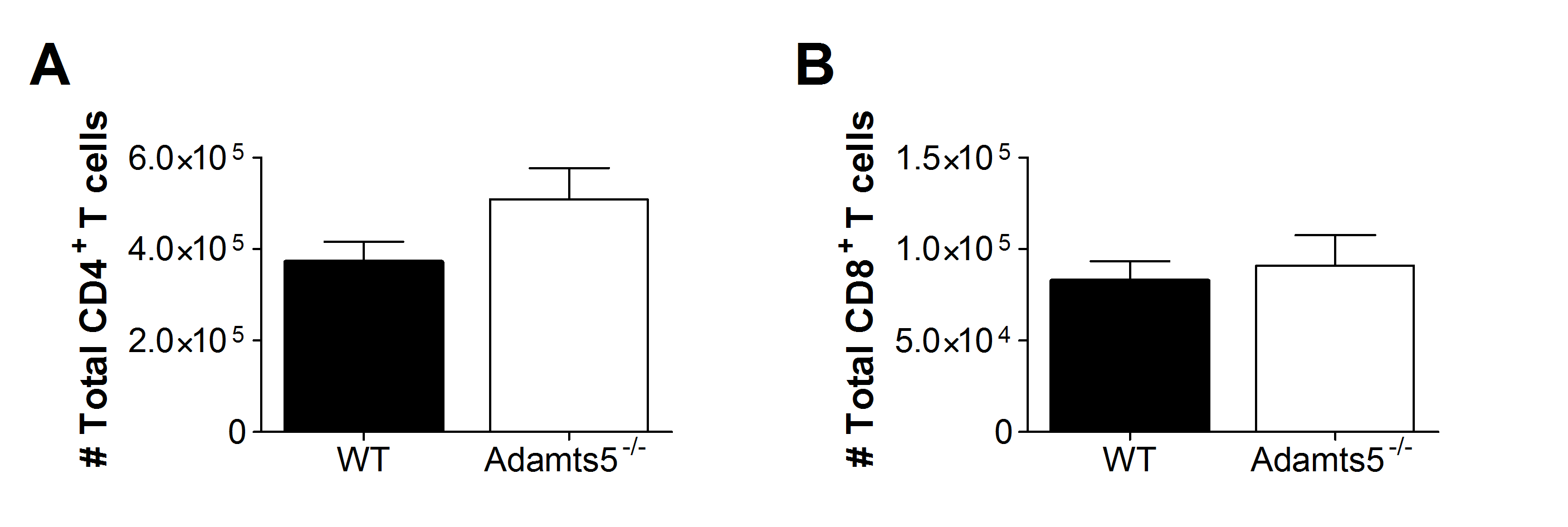

Supplement: S3 Fig — Thymus from naïve C57.BL/6 and Adamts5-/- mice were removed and immune cell subsets were characterised. Total (A) CD4+ and (B) CD8+ T cells in the thymus of naïve C57.BL/6 and Adamts5-/- mice. WT denotes C57.BL/6 mice. Results are expressed as means ± SD, and statistical significance (p < 0.05 relative to C57.BL/6) determined by Student’s t test (n = 5 mice representing three experiments). Underlying data are provided in S2 Data. (TIF) [file pbio.1002580.s005.tif]

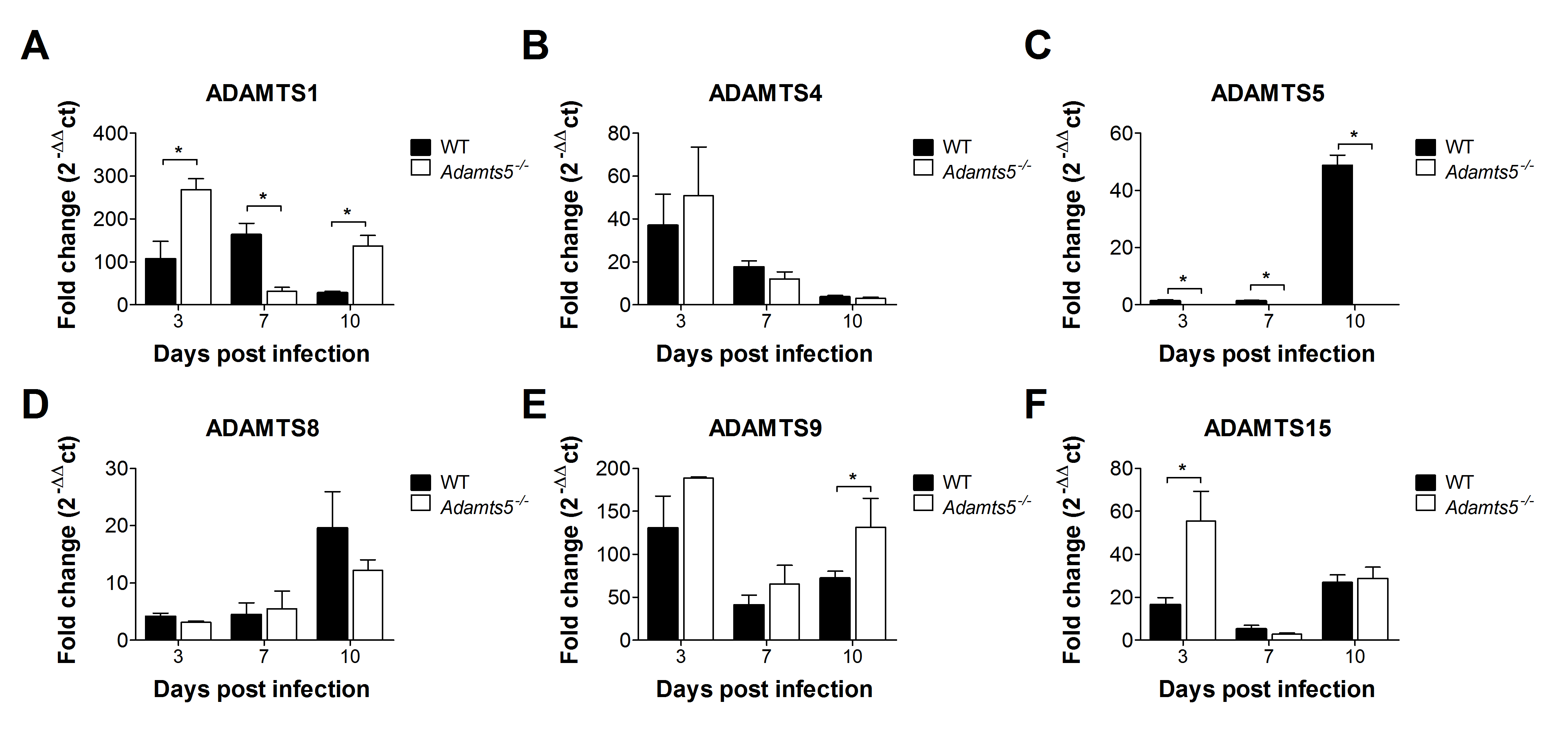

Supplement: S4 Fig — cDNA from the lungs of influenza virus infected Adamts5-/- and C57.BL/6 mice was generated and the expression of ADAMTS enzymes assessed by qRT-PCR. Expression of ADAMTS (A) 1, (B) 4, (C) 5, (D) 8, (E) 9, and (F) 15 enzymes at 0, 3, 7, and 10 d p.i. WT denotes C57.BL/6 mice. Results are expressed as means ± SD, and statistical significance (p < 0.05 relative to C57.BL/6 controls) determined by Student’s t test (n = 5 mice representing three experiments). Underlying data are provided in S2 Data. (TIF) [file pbio.1002580.s006.tif]

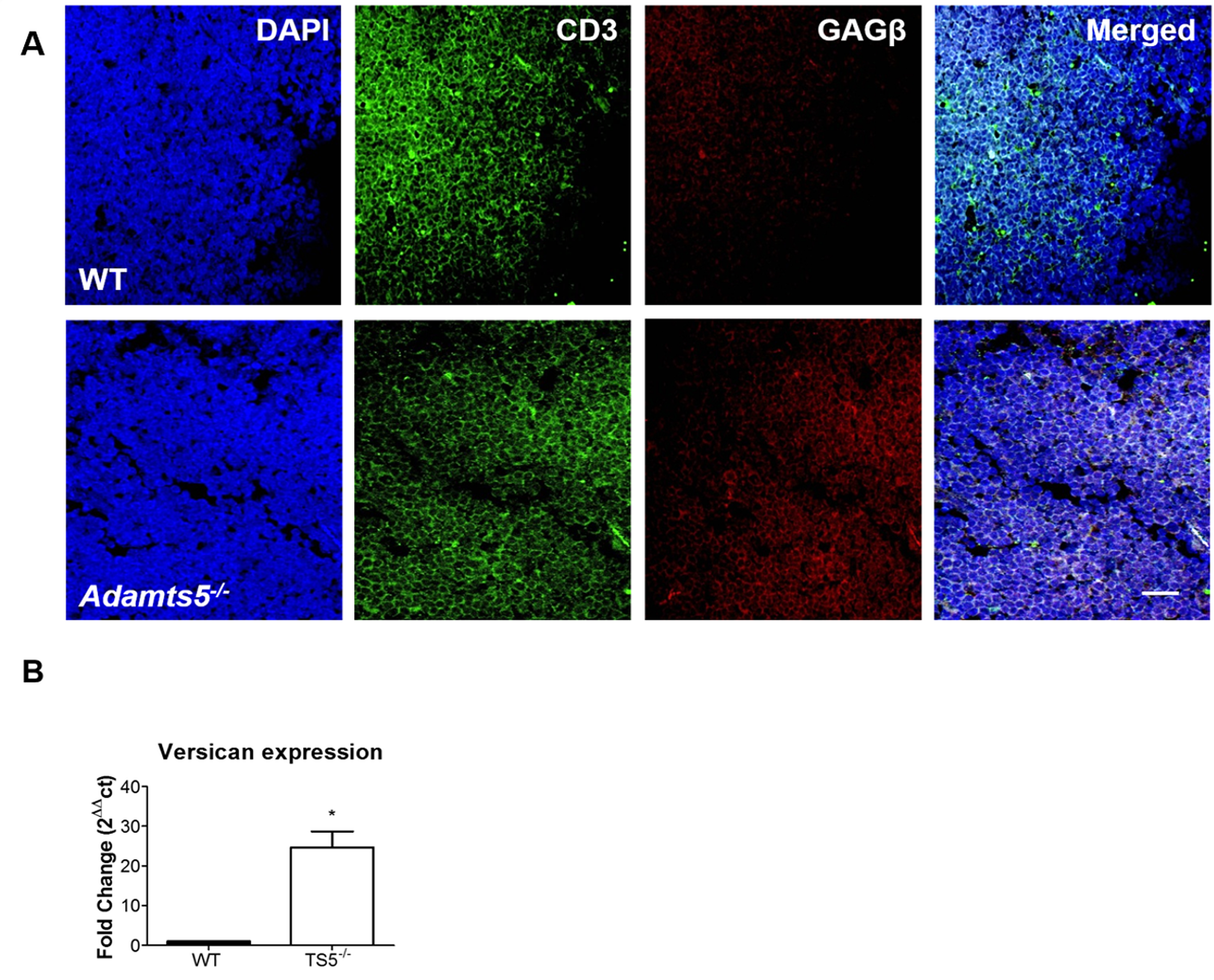

Supplement: S5 Fig — C57.BL/6 and Adamts5-/- mice were infected i.n. (104 pfu/mouse) with X31 (H3N2) influenza virus. MLNs were removed, sectioned, and stained for expression of versican (GAGβ) and CD3 (T cells). (A) Versican and T cell staining in the MLN of C57.BL/6 and Adamts5-/- mice was assessed day 7 p.i. Blue = DAPI, Red = versican (GAGβ), Green = CD3. (B) qRT-PCR of versican in the MLN. (n = 3 representing three separate experiments). Underlying data are provided in S2 Data. (TIFF) [file pbio.1002580.s007.tiff]

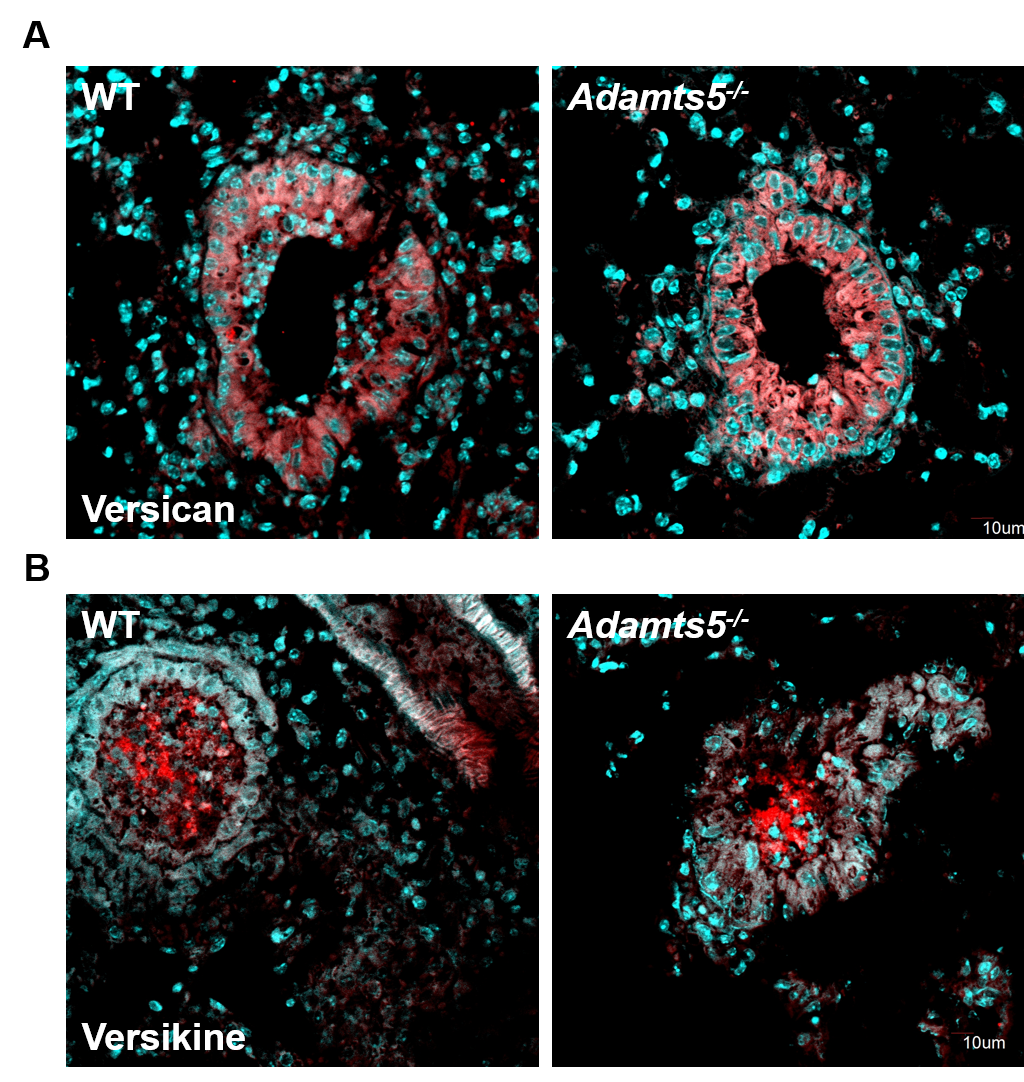

Supplement: S6 Fig — Sections of lungs from influenza virus infected Adamts5-/- and C57.BL/6 mice were assessed for the expression of versican and versikine by immunofluorescence. (A) Versican expression in the bronchiole and (B) versikine in the artery of the lung. (n = 15). WT denotes C57.BL/6 mice. (TIF) [file pbio.1002580.s008.tif]

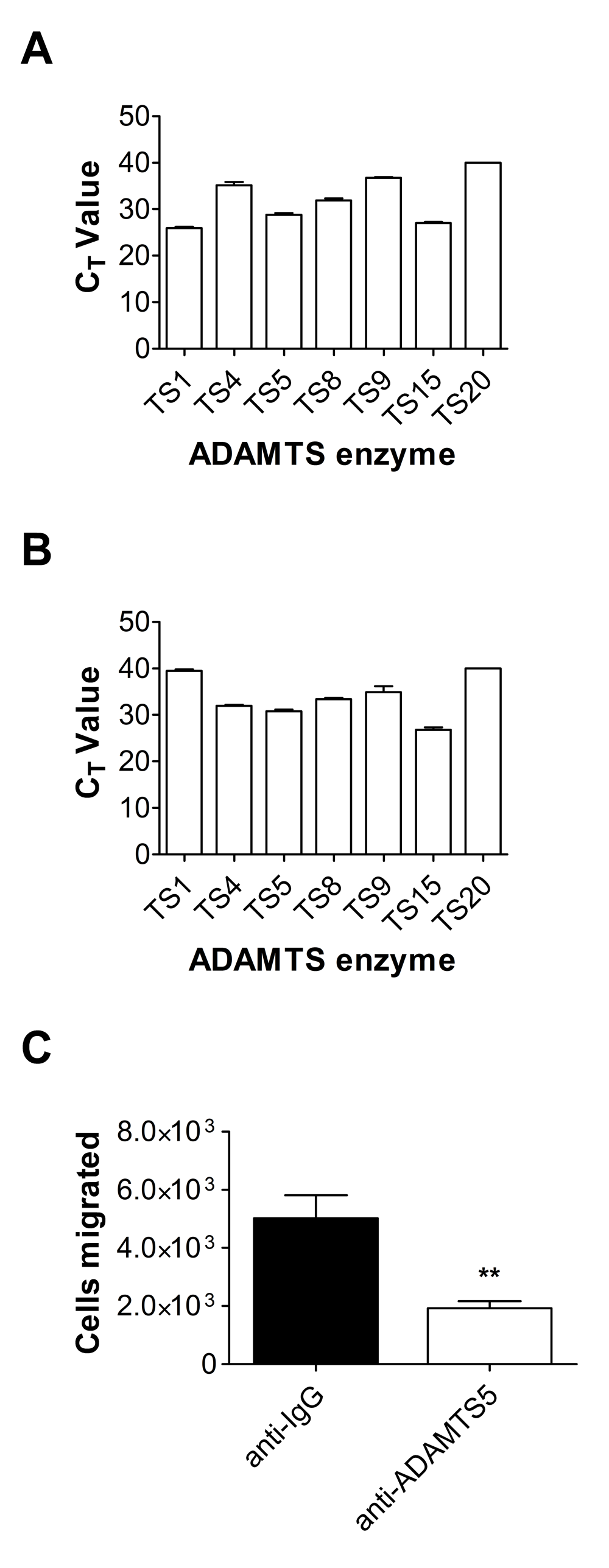

Supplement: S7 Fig — cDNA from immortalised CD4+ T cells (JURKAT cells) and peripheral blood lymphocytes was assessed for the expression of ADAMTS enzymes by qRT-PCR. Expression of ADAMTS 1, 4, 5, 8, 9, 15, and 20 enzymes in (A) JURKAT cells and (B) peripheral blood lymphocytes. (C) JURKAT cells were treated with an ADAMTS5 antibody, and migration through a versican overlay is shown by graphical representation. WT denotes C57.BL/6 mice. Results are expressed as means ± SD, and statistical significance (p < 0.05 relative to C57.BL/6 controls) determined by Student’s t test (n = 5 mice representing three experiments). Underlying data are provided in S2 Data. (TIF) [file pbio.1002580.s009.tif]

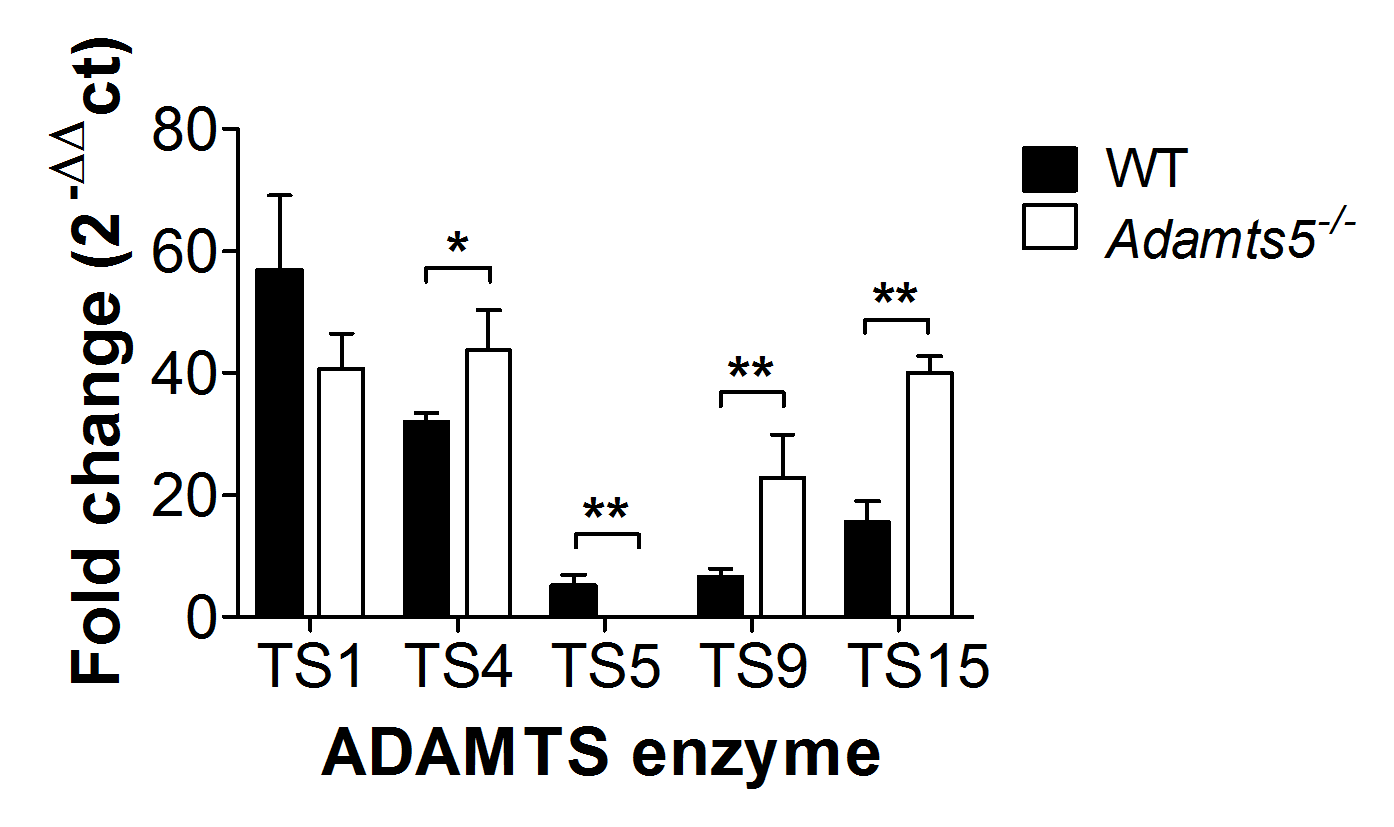

Supplement: S8 Fig — CD8+ T cells from the spleen of influenza virus infected C57.BL/6 and Adamts5-/- mice were assessed for the expression of ADAMTS enzymes (ADAMTS1, 4, 5, 9, and 15) using qRT-PCR. WT denotes C57.BL/6 mice. Results are expressed as means ± SD, and statistical significance (p < 0.05 relative to C57.BL/6 controls) determined by Student’s t test (n = 5 mice representing three experiments). Underlying data are provided in S2 Data. (TIF) [file pbio.1002580.s010.tif]

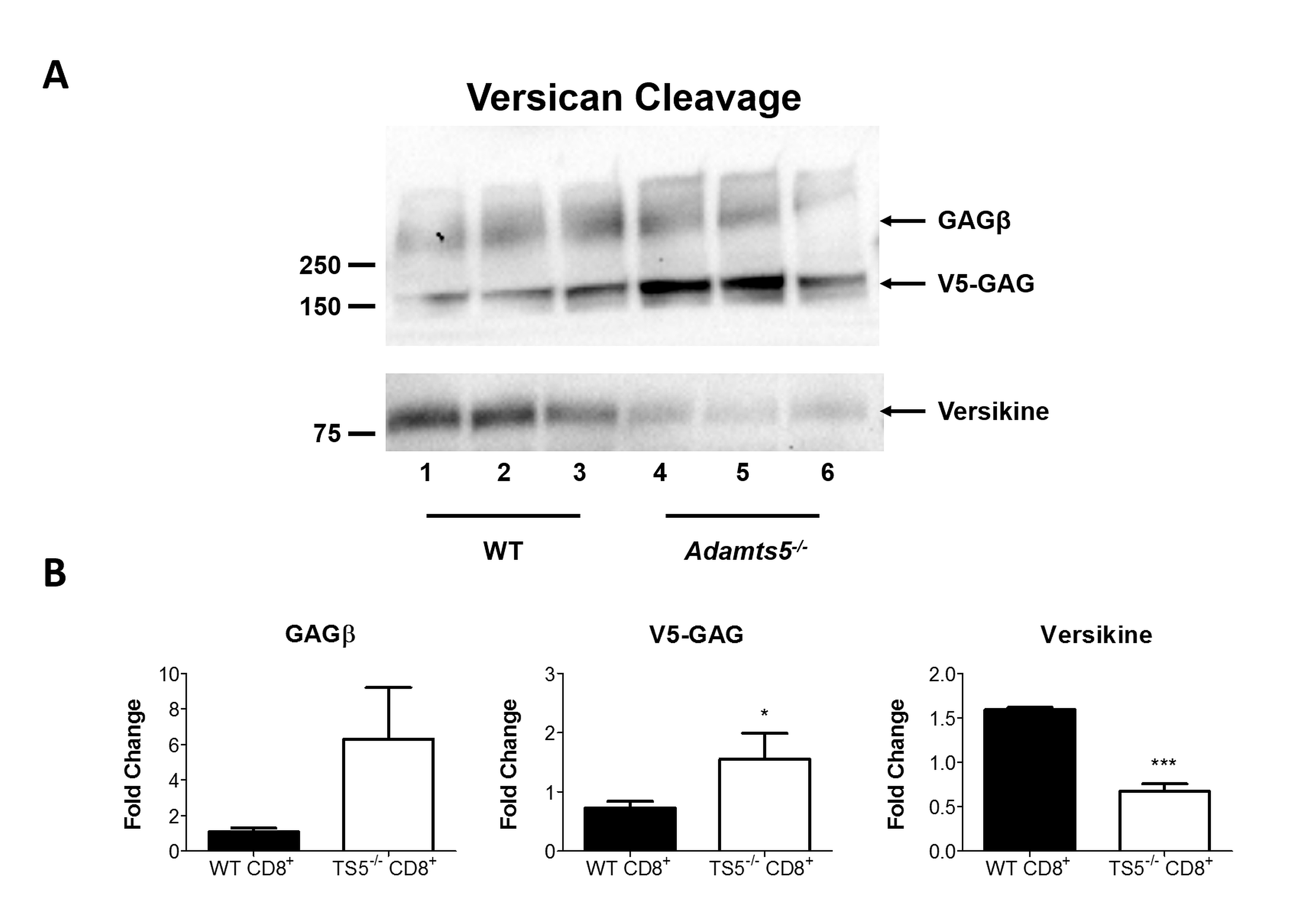

Supplement: S9 Fig — CD8+ T cells were isolated from influenza virus infected Adamts5-/- and C57.BL/6 mice and incubated with versican-conditioned media for 16 hours. Versican cleavage is shown by (A) western blot analysis and (B) densitometric quantification of protein bands using Image J software. Results are expressed as means ± SD, and statistical significance (p < 0.05 and p < 0.005 relative to C57.BL/6 controls) determined by Student’s t test (n = 5 representing three experiments). Underlying data are provided in S2 Data. (TIF) [file pbio.1002580.s011.tif]

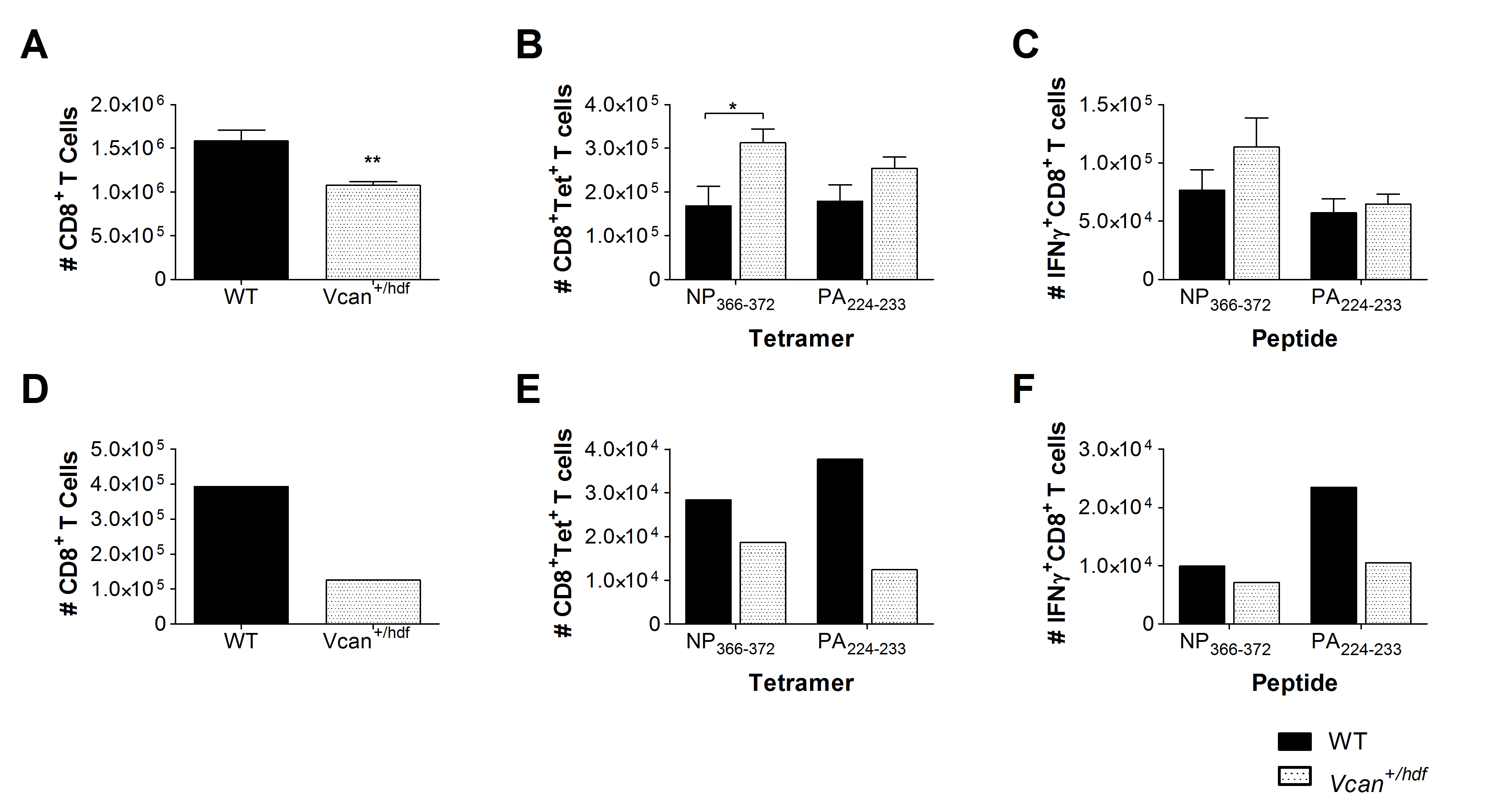

Supplement: S10 Fig — Lung tissue and MLNs were removed from influenza virus infection C57.BL/6 and Vcan+/hdf mice and processed to generate single cell suspensions at day 10 p.i. for analysis of influenza-specific immunity. (A) Total CD8+ T cell numbers were determined at day 10 p.i. in the lung. (B) Influenza-specific DbNP366-372+ CD8+ and DbPA224-233+ CD8+ tetramer positive T cells in the lung were enumerated at day 10 p.i. CD8+ T cell functionality was measured using ICS. (C) Influenza specific DbNP366-372+IFNγ+CD8+ and DbPA224-233+IFNγ+CD8+ T cell responses were characterised in the lung at day 10 p.i. (D) Total CD8+ T cell numbers were determined at day 10 p.i. from pooled MLN samples. (E) Influenza-specific DbNP366-372+ CD8+ and DbPA224-233+ CD8+ tetramer positive T cells in pooled MLN were enumerated at day 10 p.i. (F) CD8+ T cell functionality was measured using ICS to assess influenza-specific DbNP366-372+IFNγ+CD8+ and DbPA224-233+IFNγ+CD8+ T cell responses at day 10 p.i. The results are expressed as means ± SD or as pooled means (MLN data) and statistical significance (relative to C57.BL/6 mice) determined by a Student’s t test (*p ≤ 0.05, ***p ≤ 0.005 relative to C57.BL/6, n = 5 representing three individual experiments). WT denotes C57.BL/6 mice. Underlying data are provided in S2 Data. (TIFF) [file pbio.1002580.s012.tiff]

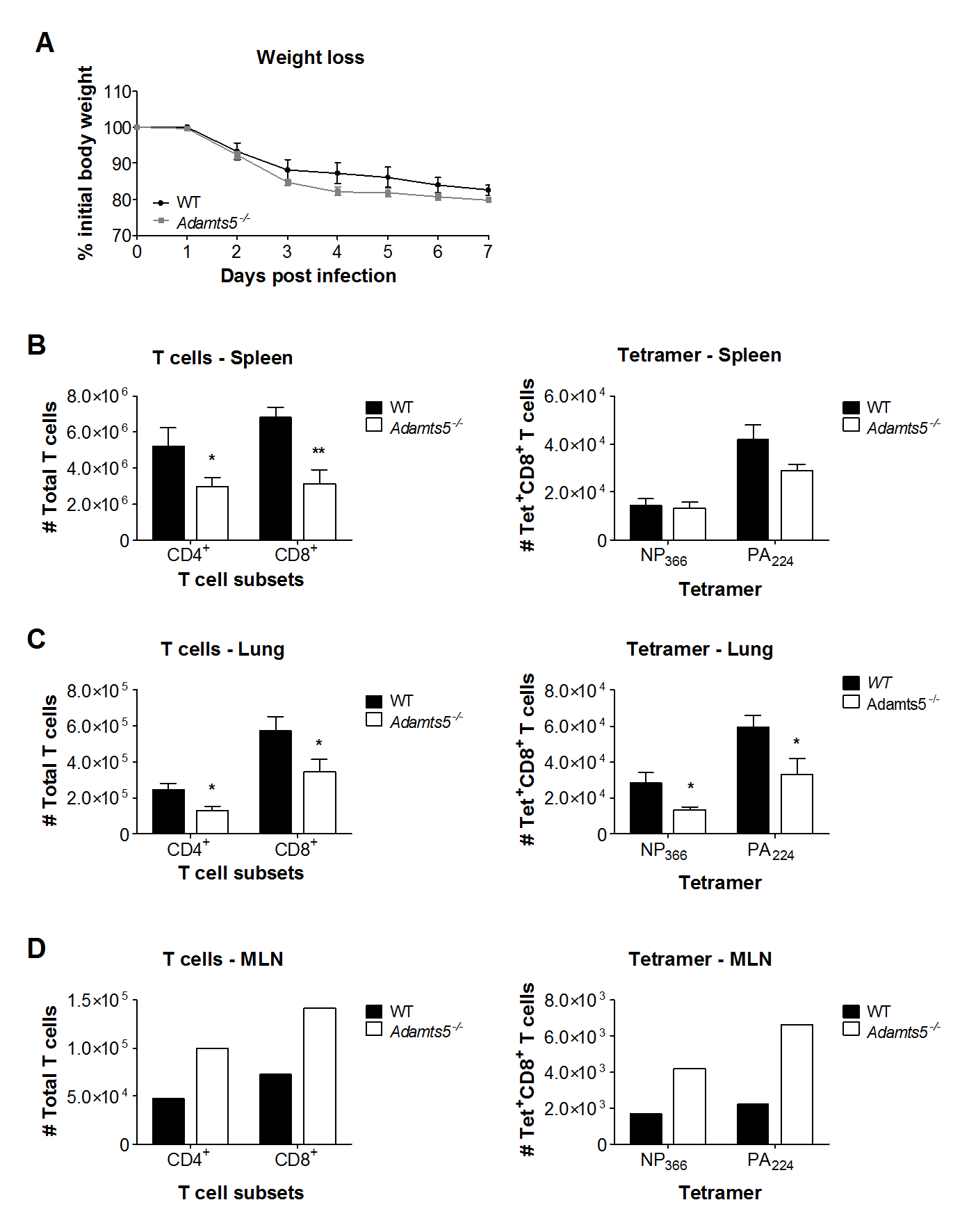

Supplement: S11 Fig — Adamts5-/- and WT mice were infected i.n with X31 (H3N2) influenza virus and spleens, lungs, and MLNs removed from C57.BL/6 and Adamts5-/- mice days 7 p.i. Single cell suspensions were then analysed for influenza-specific immunity. (A) Weight loss was calculated over the time course of infection. Total CD4+ and CD8+ T cells were enumerated in the (B) spleen, (C) lung, and (D) MLN. Influenza-specific DbNP366-372+ CD8+ and DbPA224-233+ CD8+ tetramer positive T cell numbers were also characterised in the (B) spleen, (C) lung, and (D) MLN. Lung and spleen results are expressed as means ± SD or as pooled means (MLN data), and statistical significance (relative to C57.BL/6 mice) was determined by a Student’s t test (*p ≤ 0.05, **p ≤ 0.01 relative to C57.BL/6 mice, n = 5 representing three individual experiments). WT denotes C57.BL/6 mice. Underlying data are provided in S2 Data. (TIF) [file pbio.1002580.s013.tif]
