# Supplementary figures and images for: Correction: ADAMTS5 Is a Critical Regulator of Virus-Specific T Cell Immunity
Source: PLoS Biol. 2019 Nov 6;17(11):e3000558. doi: 10.1371/journal.pbio.3000558 (PMC6834238; doi:10.1371/journal.pbio.3000558)

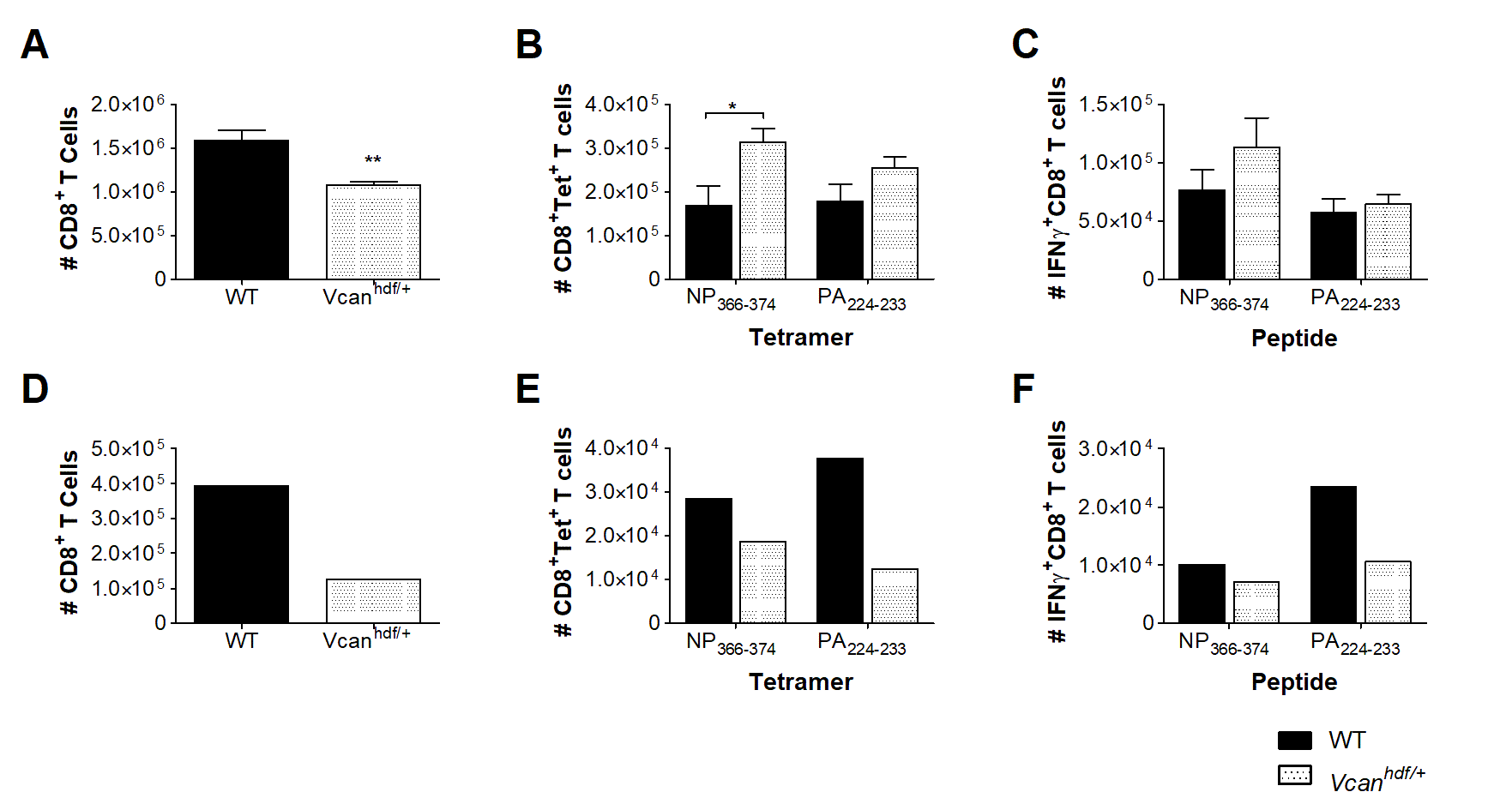

Supplement: S10 Fig — Lung tissue and MLNs were removed from influenza virus infection C57.BL/6 and Vcan+/hdf mice and processed to generate single cell suspensions at day 10 p.i. for analysis of influenza-specific immunity. (A) Total CD8+ T cell numbers were determined at day 10 p.i. in the lung. (B) Influenza-specific DbNP366-374+ CD8+ and DbPA224-233+ CD8+ tetramer positive T cells in the lung were enumerated at day 10 p.i. CD8+ T cell functionality was measured using ICS. (C) Influenza specific DbNP366-374+IFNγ+CD8+ and DbPA224-233+IFNγ+CD8+ T cell responses were characterised in the lung at day 10 p.i. (D) Total CD8+ T cell numbers were determined at day 10 p.i. from pooled MLN samples. (E) Influenza-specific DbNP366-374+ CD8+ and DbPA224-233+ CD8+ tetramer positive T cells in pooled MLN were enumerated at day 10 p.i. (F) CD8+ T cell functionality was measured using ICS to assess influenza-specific DbNP366-374+IFNγ+CD8+ and DbPA224-233+IFNγ+CD8+ T cell responses at day 10 p.i. The results are expressed as means ± SD or as pooled means (MLN data) and statistical significance (relative to C57.BL/6 mice) determined by a Student’s t test (*p ≤ 0.05, ***p ≤ 0.005 relative to C57.BL/6, n = 5 representing three individual experiments). WT denotes C57.BL/6 mice. Underlying data are provided in S2 Data. (TIF) [file pbio.3000558.s001.tif]

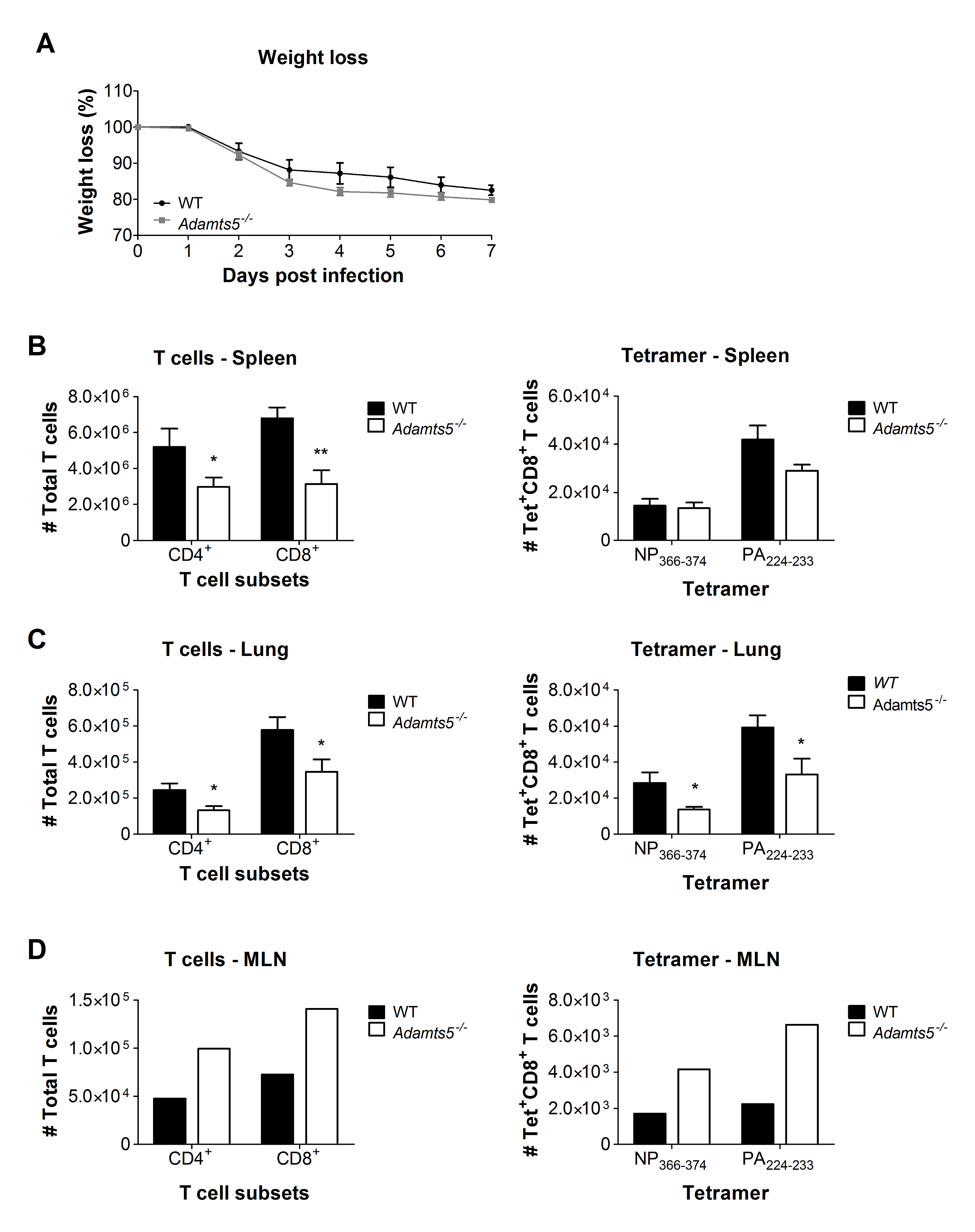

Supplement: S11 Fig — Adamts5-/- and WT mice were infected i.n with X31 (H3N2) influenza virus and spleens, lungs, and MLNs removed from C57.BL/6 and Adamts5-/- mice days 7 p.i. Single cell suspensions were then analysed for influenza-specific immunity. (A) Weight loss was calculated over the time course of infection. Total CD4+ and CD8+ T cells were enumerated in the (B) spleen, (C) lung, and (D) MLN. Influenza-specific DbNP366-374+ CD8+ and DbPA224-233+ CD8+ tetramer positive T cell numbers were also characterised in the (B) spleen, (C) lung, and (D) MLN. Lung and spleen results are expressed as means ± SD or as pooled means (MLN data), and statistical significance (relative to C57.BL/6 mice) was determined by a Student’s t test (*p ≤ 0.05, **p ≤ 0.01 relative to C57.BL/6 mice, n = 5 representing three individual experiments). WT denotes C57.BL/6 mice. Underlying data are provided in S2 Data. (TIF) [file pbio.3000558.s002.tif]
